# *NR3C1*, *LAX1*, and *RCAN3* as Circulating Epigenetic Biomarkers for Prognosis and Chemotherapy Response Prediction in Metastatic Pancreatic Cancer

**DOI:** 10.1002/mco2.70682

**Published:** 2026-03-22

**Authors:** Pablo Cano‐Ramírez, Marta Toledano‐Fonseca, María Teresa Cano‐Osuna, Nerea Herrera‐Casanova, Emilio Carrillo‐Pecero, Antonio Rodríguez‐Ariza, Enrique Aranda, María Victoria García‐Ortiz

**Affiliations:** ^1^ University of Córdoba Córdoba Spain; ^2^ Maimonides Biomedical Research Institute of Cordoba (IMIBIC) Córdoba Spain; ^3^ Reina Sofía University Hospital Córdoba Spain; ^4^ Cancer Network Biomedical Research Centre (CIBERONC) Madrid Spain; ^5^ Medical Oncology Department Reina Sofía University Hospital Córdoba Spain

**Keywords:** DNA methylation and response prediction, liquid biopsy, pancreatic ductal adenocarcinoma

## Abstract

Pancreatic cancer remains highly lethal, largely due to late diagnosis and limited efficacy of treatments. Improving first‐line treatment selection and patient monitoring requires novel, non‐invasive biomarkers beyond carbohydrate antigen 19‐9 (CA19‐9) and imaging. This study investigates epigenetic biomarkers from liquid biopsy with prognostic and predictive potential in metastatic pancreatic ductal adenocarcinoma (PDAC; mPDAC). Genome‐wide methylation profiling of cell‐free DNA (cfDNA) from healthy individuals and stage IV mPDAC patients identified 13 gene‐associated CpG sites with significantly altered methylation patterns. ddPCR validation confirmed consistent methylation differences in lymphocyte transmembrane adaptor 1 (*LAX1*), nuclear receptor subfamily 3 group C member 1 (*NR3C1*), and *RCAN3* between healthy and patient groups. Elevated *LAX1* and *RCAN3* methylation and reduced *NR3C1* methylation at diagnosis were associated with poor prognosis and correlated with high‐risk circulating biomarker profiles, including CA19‐9 levels, *RAS* MAF (mutant allele fraction), cfDNA concentration, and cfDNA fragmentation. Notably, baseline *NR3C1* methylation levels predicted response to first‐line FOLFIRINOX‐based treatment with an acceptable 75% sensitivity and a high specificity of 92.86%. These findings highlight the clinical significance of cfDNA methylation as a minimally invasive biomarker source, emphasizing *LAX1*, *NR3C1*, and *RCAN3* as prognostic biomarkers in mPDAC. Specifically, baseline *NR3C1* methylation emerges as a promising predictor of treatment response, supporting personalized therapeutic strategies in mPDAC.

## Graphical abstract

Clinical utility of liquid biopsy‐based epigenetic profiling in PDAC management. Aberrant methylation of *LAX1*, *RCAN3*, and *NR3C1* was identified in cfDNA from mPDAC patients, highlighting their potential as non‐invasive epigenetic biomarkers. Methylation levels of these genes showed prognostic value, supporting their relevance for patient stratification and disease monitoring. Notably, *NR3C1* methylation emerged as a predictive biomarker of first‐line treatment response, with high circulating levels—potentially reflecting low tumor expression—associated with improved outcomes under FOLFIRINOX‐based therapies. Some elements of this figure were sourced from Servier Medical Art (https://smart.servier.com/).

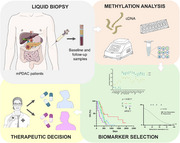

## 1 Background

Pancreatic cancer is one of the deadliest malignancies, with a 5‐year survival rate of 8.2% [1]. Risk factors comprise family history, obesity, diabetes, smoking, and high alcohol consumption [2, 3]. Specifically, pancreatic ductal adenocarcinoma (PDAC) represents nearly 90% of pancreatic malignancies [4] and is characterized with dismal prognosis due to the lack of specific symptoms in the early stages of the disease [5], the absence of effective diagnostic methods, and the low efficacy of current treatments [6]. As a result, most patients (> 75%) are diagnosed at an advanced or metastatic stage, and only 15%–20% are candidates for curative surgery. Even following resection, relapse occurs early, resulting in a median survival of 10–20 months [7]. In metastatic cases, median overall survival (OS) from diagnosis is approximately 4.6 months [8].

FOLFIRINOX (a combination of fluorouracil, irinotecan, leucovorin, and oxaliplatin) and gemcitabine are the primary first‐line treatments for metastatic PDAC patients. FOLFIRINOX treatment has been reported to offer improved survival over gemcitabine‐based regimens, although response is heterogeneous and not all patients benefit [9]. Indeed, as a more aggressive therapy with higher toxicity (Grade 3/4), FOLFIRINOX‐based therapy is reserved for patients with a good ECOG (Eastern Cooperative Oncology Group) performance status [10, 11].

In recent years, advances in the analysis of tumor tissues and cell lines have improved our understanding of pancreatic cancer and led to the identification of distinct molecular subtypes [12]. Notably, Moffitt et al. proposed a gene expression‐based classification that accounted for stromal contamination in PDAC samples, identifying two main subtypes: basal‐like, associated with poorer survival, and classical, linked to more favorable outcomes [13]. Despite these advances, molecular classification of pancreatic cancer remains in its early stages, and there is currently no consensus on its clinical implementation for patient management [14].

Currently, carbohydrate antigen 19‐9 + remains the only blood‐based biomarker used to guide clinical management in pancreatic cancer, though it has relatively low sensitivity (79%) and specificity (82%) [15]. While CA19‐9 levels are linked to survival in mPDAC patients [16], there is no consensus on how changes in CA19‐9 levels should be interpreted during the disease progression [17]. Consequently, effective biomarkers are required to inform prognosis at diagnosis, as well as to support ongoing monitoring aimed at detecting early recurrence after surgical resection or treatment failure in advanced cases.

Compared to tumor biopsies, analysis of cell‐free DNA (cfDNA) in liquid biopsies provides a more comprehensive overview of the tumor landscape, as cfDNA is released from multiple tumor regions and metastatic sites, thereby capturing tumor heterogeneity [18]. In addition, liquid biopsy offers higher sensitivity than conventional imaging techniques, enabling the detection of tumors composed of as few as 50 million malignant cells, which remain undetectable by computed tomography (CT) that typically requires tumor burdens exceeding one billion cells (approximately 7–10 mm) [19].

Importantly, the minimally invasive nature of liquid biopsy allows for sequential sampling and longitudinal monitoring, facilitating the dynamic assessment of cfDNA concentration and fragmentation [20], as well as the emergence of genetic or epigenetic alterations associated with tumor evolution and treatment response [21].

In this regard, our group has demonstrated the utility of the *RAS* mutant allele fraction (MAF) and *NPTX2* methylation dynamics in monitoring pancreatic disease evolution in advanced stage, both showing a strong correlation with CT scan results [20, 22]. Notably, dynamic variations in *NPTX2* methylation anticipated disease progression detected by CT scans and CA19‐9 measurements [22].

However, studies identifying epigenetic biomarkers that enable a personalized therapeutic approach for pancreatic cancer are limited and often conducted in cell lines [23, 24, 25].

In the present study, we have identified epigenetic biomarkers in cfDNA that have the potential to guide first‐line treatment in patients with metastatic pancreatic cancer and monitor disease progression. Using a methylation array to profile cfDNA from both healthy individuals and stage IV pancreatic cancer patients, three differentially methylated genes were identified. Subsequent validation confirmed this cfDNA methylation profile and demonstrated that circulating nuclear receptor subfamily 3 group C member 1 (*NR3C1*) methylation could effectively stratify patients based on their response to first‐line FOLFIRINOX treatment, offering valuable insights into disease progression and correlating with treatment outcomes.

## 2 Results

### 2.1 Analysis of Patient Clinicopathological Characteristics

The clinicopathological characteristics of the patients are summarized in Table 1. Among the 69 patients included in the study, 28 were female, and 41 were male, with ages ranging from 48 to 84 years and a median age of 65. The primary tumor locations varied, with occurrences in the head (29%), tail (30.4%), and body (37.7%) and two cases where the location was undetermined. All patients presented with mPDAC, and liver metastases were observed in 87% of cases. Most patients (87%) had a good baseline performance status, classified as ECOG 0–1, and gemcitabine‐based regimens were the most commonly administered treatment, used in 56.5% of cases. *RAS* mutations were detected in plasma in 79.7% of patients. The prevalence of risk factors was as follows: cigarette smoking in 52.2%, alcohol consumption in 42%, diabetes mellitus in 34.8%, and arterial hypertension in 50.7% of the patients.

**TABLE 1:** Clinicopathological characteristics of patients.

Patient characteristics	Number of patients (*n* = 69)	Percentage
**Age**		
≤ 65 years	41	59.4
> 65 years	28	40.6
**Sex**		
Male	41	59.4
Female	28	40.6
**Stage**		
IV	69	100
**ECOG**		
0	32	46.4
1	28	40.6
2/3	9	13
**First‐line treatment**		
FOLFIRINOX‐based	23	33.3
Gemcitabine‐based^a^	39	56.5
Others^b^	5	7.2
No treatment	2	2.9
**Tobacco smoking**		
Never	32	46.4
< 1 pack/day	16	23.2
> 1 pack/day	20	29.0
N/A	1	1.4
**Alcohol intake**		
Never	38	55.1
Ocassional	15	21.7
Current daily	14	20.3
N/A	2	2.9
**Diabetes**		
No	45	65.2
Yes	24	34.8
**Hypertension**		
Yes	35	50.7
No	34	49.3
**Primary tumor location**		
Head	20	29
Tail	21	30.4
Body	26	37.7
N/A	2	2.9
**Metastatic lesions location**		
Non‐hepatic	9	13
Hepatic	60	87
***RAS* status in plasma**		
*RAS* mutated	55	79.7
*RAS* wild type	14	20.3

^a^ Gemcitabine‐based treatment includes gemcitabine monotherapy and gemcitabine Abraxane.

^b^ Others first‐line treatment includes cisplatin and gemcitabine with FOLFOX.

Disease progression occurred in 67 out of 69 patients, and by the conclusion of the study, 63 had reached exitus, while six remained alive. No differences in OS or progression‐free survival (PFS) were observed when patients were stratified by age, tobacco smoking, occasional alcohol consumption, diabetes mellitus, arterial hypertension, or primary tumor location (Table 2). A longer PFS was observed in patients with non‐hepatic metastases (*p* = 0.015), whereas PFS was significantly shorter in daily drinkers, compared to those patients who never consumed alcohol (*p* = 0.040). FOLFIRINOX‐based first‐line treatment, female gender, and ECOG 0 status were associated with better OS (*p* = 0.004, *p* = 0.014, and *p* = 0.030 vs. ECOG 1 and *p =* 0.009 vs. ECOG 2, respectively) and PFS (*p* = 0.005, *p* = 0.003, and *p* = 0.024 vs. ECOG 1 and *p =* 0.005 vs. ECOG 2, respectively).

**TABLE 2:** Overall survival (OS) and progression‐free survival (PFS) analysis of clinicopathological characteristics.

Variables	Median OS	OS		Median PFS	PFS	
	(days)	HR (95% CI)	*p*	(days)	HR (95% CI)	*p*
**Age**						
≤ 65 years^a^	274	0.974	0.918	180	0.873	0.590
> 65 years	372	(0.591–1.606)		204	(0.527–1.447)	
**Sex**						
Male^a^	264	1.849	0.014	133	2.085	0.003
Female	440	(1.128–3.031)		295	(1.270–3.423)	
**ECOG**						
0^a^	419			232		
1	266	0.567 (0.324–0.994)	0.030	163	0.555 (0.316–0.975)	0.024
2 or 3	142	0.389 (0.140–1.084)	0.009	65	0.365 (0.127–1.044)	0.005
**First‐line treatment**						
FOLFIRINOX‐based^a^	482	0.459	0.004	299	0.457	0.005
Gemcitabine‐based^b^	264	(0.272–0.775)		133	(0.271–0.773)	
**Metastatic location**						
Hepatic^a^	292	2.012	0.068	167	2.482	0.015
Non‐hepatic	435	(1.086–3.726)		391	(1.388–4.437)	
**Tobacco smoking**						
Never^a^	273			187		
< 1 pack/day	370	0.897 (0.468–1.720)	0.732	200	0.964 (0.507–1.832)	0.909
> 1 pack/day	351	0.836 (0.463–1.511)	0.534	156	0.799 (0.441– 1.450)	0.443
**Alcohol intake**						
Never^a^	293			204		
Ocassional	397	0.816 (0.432–1.542)	0.504	203	0.752 (0.393– 1.440)	0.350
Current daily	227	0.586 (0.279–1.228)	0.088	99	0.524 (0.243– 1.129)	0.040
**Hypertension**						
No^a^	338	0.918	0.729	196	1.007	0.977
Yes	345	(0.560–1.504)		174	(0.615–1.650)	
**Diabetes**						
No^a^	336	1.157	0.560	180	1.141	0.616
Yes	349	(0.696–1.923)		199	(0.686–1.897)	
**Primary tumor location**						
Head^a^	292	0.800	0.422	187	0.681	0.153
Body/tail	339	(0.469–1.363)		174	(0.405–1.146)	

^a^ Reference category for analysis.

^b^ Gemcitabine‐based treatment includes gemcitabine monotherapy and gemcitabine Abraxane.

### 2.2 Differentially Methylated Regions in cfDNA From Healthy Individuals and Patients With mPDAC

**FIGURE 1:**
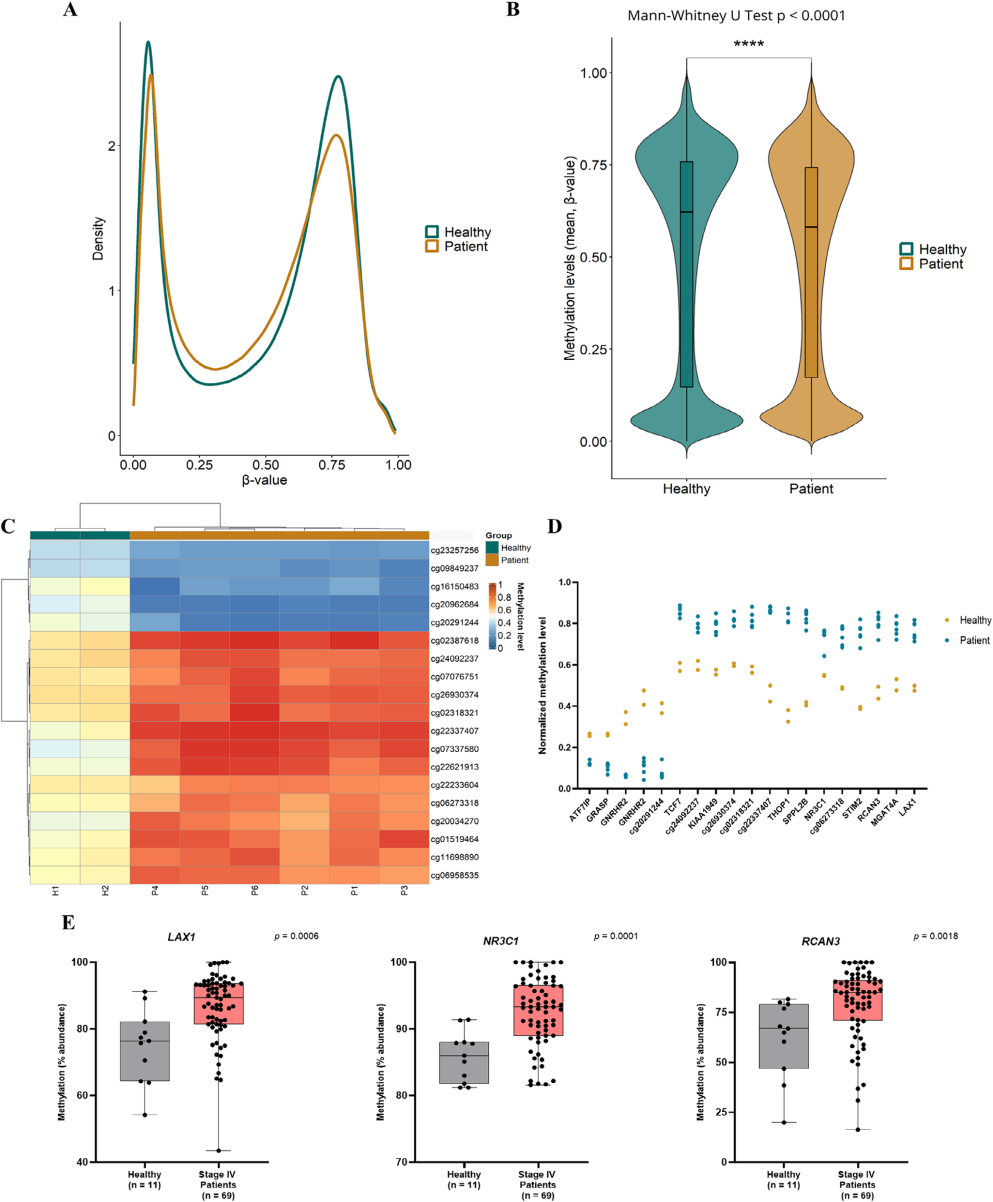
Landscape of DNA methylation in cfDNA from healthy individuals and metastatic PDAC (mPDAC) patients. (A) Density plot displaying the characteristic bimodal distribution of methylation β‐values in the two groups based on EPIC array analysis data. (B) Violin plot for the overall beta value distribution, showing the mean methylation values. ****p < 0.0001. (C) Heat map and hierarchical clustering analysis of the EPIC array based on the 19 differentially methylated CpGs (adj. *p* < 005) between healthy controls (H1 and H2) and mPDAC patients at diagnosis (P1‐P6). Methylation levels were scaled from 0 to 1. (D) Normalized methylation levels of the 19 most significant differentially methylated CpGs between healthy individuals and mPDAC patients. (E) Relative CpG methylation levels of *LAX1*, *NR3C1*, and *RCAN3* genes detected by ddPCR in plasma cfDNA from healthy individuals (*n* = 11) and mPDAC patients (*n* = 69). Data are mean ± SD.

To thoroughly investigate the differences in methylation patterns between healthy and mPDAC conditions, a methylation array was performed using the Illumina EPIC 850k BeadChip, with cfDNA extracted from two healthy individuals and six mPDAC individuals at the time of diagnosis as the starting material. The β‐values in the two groups exhibited characteristic bimodal distributions (Figure 1A), with mPDAC patients, on average, showing lower methylation levels than healthy individuals (*p* < 0.0001; Figure 1B). Additionally, 19 differentially methylated CpG positions (adjusted *p*‐value ≤ 0.05) were identified, with 14 hypermethylated and five hypomethylated in stage IV pancreatic cancer patients, compared to healthy controls (Figure 1C,D). Most of these CpG sites (68%) are located within genic regions, with more than half situated in CpG islands or their flanking regions (Table 3).

**TABLE 3:** Top 19 differentially methylated CpG positions between healthy individuals and metastatic pancreatic ductal adenocarcinoma (mPDAC) patients.

Probe ID	Methylation difference in mPDAC	Adj *p*‐value	Chromosome	Probe position	Associated gene	Gene feature
cg07337580	**0.58**	0.0046	19	2809333	***THOP1***	Body‐shore
cg22621913	**0.49**	0.0065	19	2352414	***SPPL2B***	Body‐island
cg02387618	**0.27**	0.0342	5	133453480	***TCF7***	Body‐shelf
cg22337407	**0.41**	0.0342	1	11046185		IGR
cg20962684	**−0.34**	0.0389	1	145515327	***GNRHR2***	Body‐shore
cg06273318	**0.33**	0.0432	2	64553381		IGR
cg22233604	**0.25**	0.0432	5	142729377	***NR3C1***	Body‐opensea
cg26930374	**0.23**	0.0432	14	23013826		IGR
cg24092237	**0.23**	0.0432	14	22995133		IGR
cg16150483	**−0.44**	0.0432	1	145515286	***GNRHR2***	Body‐shore
cg02318321	**0.26**	0.0432	14	23007177		IGR
cg11698890	**0.34**	0.0432	2	99254310	***MGAT4A***	Body‐opensea
cg20291244	**−0.39**	0.0432	11	11820535		IGR
cg06958535	**0.35**	0.0457	1	203734478	***LAX1***	First exon
cg07076751	**0.27**	0.0457	6	30647539	***KIAA1949***	Body‐opensea
cg23257256	**−0.18**	0.0457	12	14538000	***ATF7IP***	5'UTR‐opensea
cg01519464	**0.40**	0.0457	1	24861818	***RCAN3***	3'UTR‐island
cg20034270	**0.46**	0.0457	4	26886485	***STIM2***	Body‐opensea
cg09849237	**−0.20**	0.0457	12	52404250	***GRASP***	Body‐shelf

Abbreviation: IGR: intergenic region.

Among the 13 differentially methylated CpG positions associated with genes in patients with mPDAC, compared to healthy individuals (adjusted *p*‐value ≤ 0.05), we focused on those hypermethylated sites to perform a first validation using droplet digital PCR (ddPCR). This approach employed methylation controls and probes specifically targeting CpG positions identified as differentially methylated in the array analysis. Only those CpG positions showing a relative methylation abundance ≥ 95% in methylated and ≤ 5% in unmethylated control DNA samples (lymphocyte adaptor 1 [*LAX1*], *RCAN3*, *NR3C1*, and *KIAA1949;* Table S1) were selected for further analysis.

These four targets were validated by ddPCR in the study's extended cohort of healthy individuals and mPDAC patients. This analysis confirmed statistically significant differences in methylation levels for *LAX1* (*p* = 0.0006), *NR3C1* (*p* = 0.0001), and *RCAN3* (*p* = 0.0018) between the two groups (Figure 1E), whereas no significant differences were observed for *KIAA1949* (*p* = 0.5055; Figure S1). Considering the lower mean age of healthy controls compared to patients, we conducted additional analyses to determine whether age might confound the observed group differences in methylation levels of *LAX1*, *RCAN3*, and *NR3C1*. We applied multiple linear regression models including group (healthy vs. patient) and age as covariates. The analysis confirmed that group remained a significant predictor of methylation levels for *LAX1* (*p* = 0.0073) and *NR3C1* (*p* = 0.0005), while *RCAN3* showed a borderline association (*p* = 0.0612; data not shown). In all cases, age did not exert a significant effect (*LAX1 p* = 0.9514, *RCAN3 p* = 0.7465, and *NR3C1 p* = 0.2049), indicating that the methylation differences between groups are not driven by age (Figure S2).

### 2.3 Basal Circulating Methylation Levels of *LAX1*, *RCAN3*, and *NR3C1* Are Associated With Other Circulating Tumor Biomarkers in mPDAC

**FIGURE 2:**
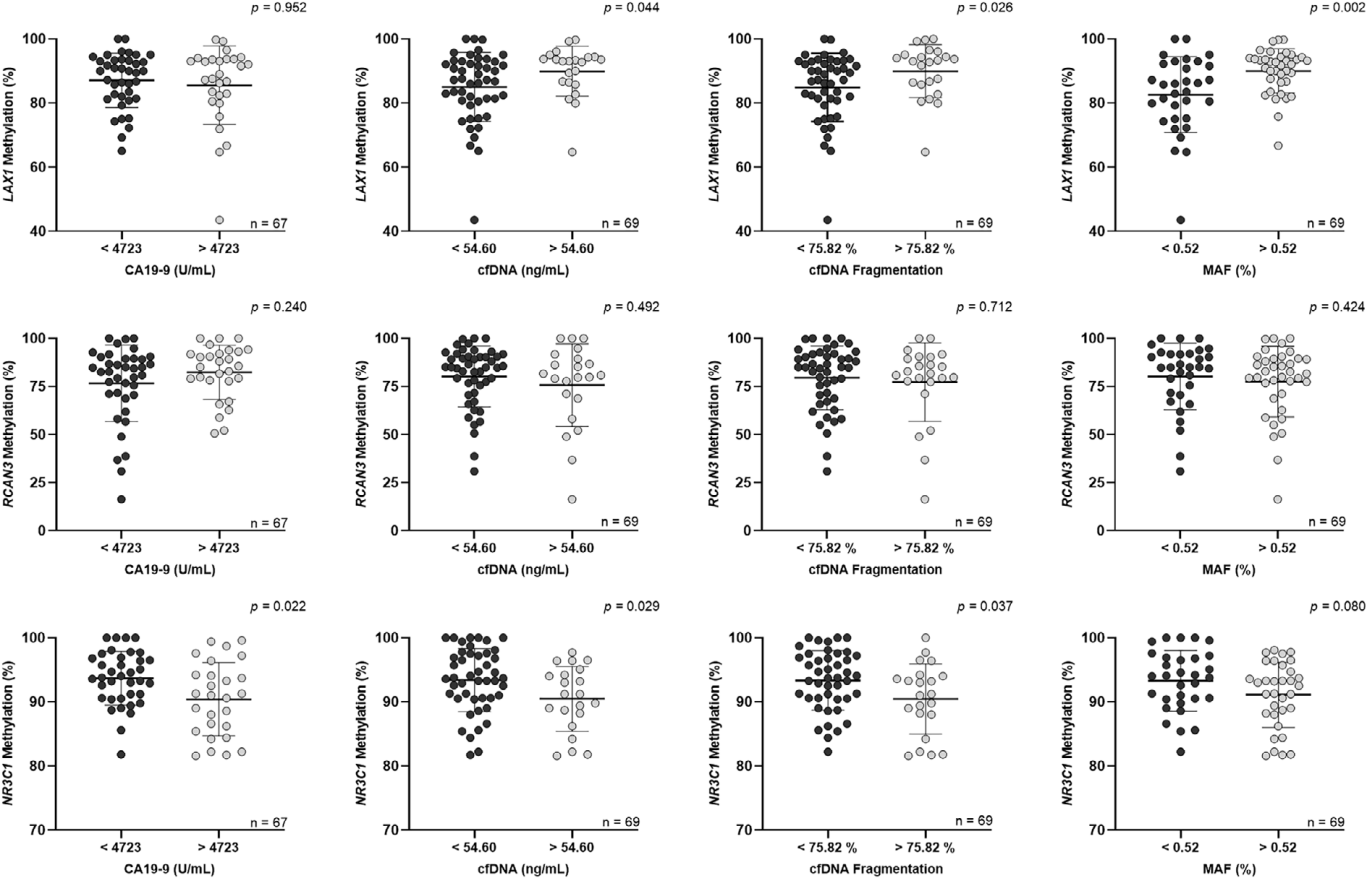
Association of basal circulating *LAX1*, *RCAN3*, and *NR3C1* methylation levels with other circulating tumor biomarkers in mPDAC. *LAX1, RCAN3*, and *NR3C1* methylation levels in plasma at diagnosis according to CA19‐9 levels, cfDNA concentration, cfDNA fragmentation, and *RAS* MAF. Cut‐off values for each biomarker were: 0.52 % for *RAS* MAF, 4723 U/mL for CA19‐9, 54.60 ng/mL for cfDNA concentration, and 75.82 % for cfDNA fragmentation. Data are mean ± SD.

We examined the potential association between circulating methylation levels of *LAX1*, *RCAN3*, and *NR3C1* and other tumor‐related circulating biomarkers, including *RAS* mutational status and *RAS* MAF in cfDNA, CA19‐9 levels or cfDNA concentration and fragmentation, in all 69 liquid biopsy samples obtained at diagnosis (Figure 2).

For this analysis, cut‐off values according to OS for each biomarker were determined using the R2 platform. The *LAX1* methylation levels were significantly elevated in those samples with a higher *RAS* MAF (92.10% vs. 84.65%, *p =* 0.002), higher cfDNA concentration (93.05% vs. 87.20%, *p =* 0.044), and higher cfDNA fragmentation (93% vs. 87.20%, *p =* 0.026). Conversely, elevated *NR3C1* methylation was significantly associated with lower CA19‐9 levels (93.80% vs. 91.00%, *p* = 0.022), lower cfDNA concentration (93.65% vs. 91.20%, *p* = 0.029), reduced cfDNA fragmentation (93.70% vs. 91.10%, *p* = 0.037), and lower *RAS* MAF (93.29% vs. 91.10%, *p* = 0.080). Accordingly, circulating *LAX1* methylation levels showed a significant positive correlation with cfDNA concentration, cfDNA fragmentation and *RAS* MAF, whereas *NR3C1* methylation levels were negatively correlated with CA19‐9 levels, cfDNA concentration and cfDNA fragmentation (Figure S3).

Of note, cfDNA concentration and fragmentation were significantly higher in mPDAC patients, compared to healthy individuals (Figure S4). Finally, no significant association was observed between *RCAN3* methylation levels and any of the other circulating biomarkers studied.

### 2.4 Circulating Biomarkers Showed Strong Prognostic Value in mPDAC

**FIGURE 3:**
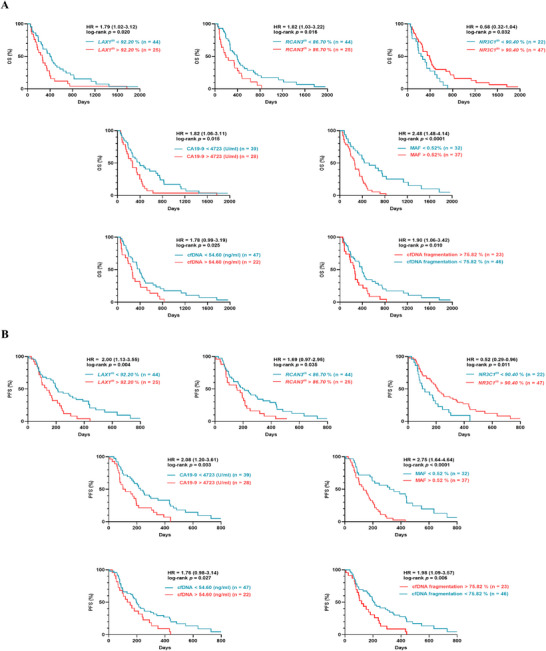
Circulating biomarkers show strong prognostic value in mPDAC plasma. (A) Overall survival (OS) and (B) progression‐free survival (PFS) according to *LAX1* methylation, *RCAN3* methylation, *NR3C1* methylation, CA19‐9 levels, *RAS* MAF, cfDNA concentration, and cfDNA fragmentation.

To evaluate the prognostic potential of circulating levels of *LAX1*, *RCAN3*, and *NR3C1* methylation, along with other biomarkers, OS and PFS were analyzed using Kaplan–Meier curves with cut‐off values from R2 platform (Table 4 and Figure 3).

**TABLE 4:** Analysis of OS and PFS according to basal circulating biomarkers in mPDAC patients.

Variable	At diagnosis (%) *n* = 69	Median OS (days)	OS HR (95% CI)	*p*	Median PFS (days)	PFS HR (95% CI)	*p*
**CA19‐9**	**(*n* = 67)**						
> 4723 (U/mL)^a^	28 (42%)	266	1819	0.014	110	2078	0.0025
≤ 4723 (U/mL)	39 (58%)	391	(1.063–3.113)		226	(1.196–3.608)	
**cfDNA concentration**							
> 54.60 (ng/mL)^a^	22 (32%)	254	1778	0.025	143	1755	0.027
≤ 54.60 (ng/mL)	47 (68%)	391	(0.990–3.192)		196	(0.980–3.144)	
***RAS* status in plasma**							
*RAS* mutated^a^	55 (80%)	271	3021	0.0001	164	3148	< 0.0001
*RAS* wild‐type	14 (20%)	658	(1.815–5.027)		440	(1.878–5.278)	
**MAF**							
> 0.52 %^a^	37 (54%)	264	2478	< 0.0001	133	2753	< 0.0001
≤ 0.52 %	32 (46%)	479	(1.484–4.139)		319	(1.635–4.636)	
**cfDNA fragmentation**							
> 75.82 %^a^	23 (33%)	260	1906	0.010	119	1975	0.006
≤ 75.82 %	46 (67%)	397	(1.062–3.422)		213	(1.094–3.565)	
***LAX1* methylation**							
> 92.20 %^a^	25 (36%)	260	1787	0.020	133	2002	0.004
≤ 92.20 %	44 (64%)	419	(1.024–3.119)		213	(1.130–3.549)	
***RCAN3* methylation**							
> 86.70 %^a^	25 (36%)	194	1824	0.016	157	1690	0.035
≤ 86.70 %	44 (64%)	395	(1.033–3.220)		204	(0.967–2.953)	
***NR3C1* methylation**							
> 90.40 %^a^	47 (68%)	391	0.578	0.032	211	0.524	0.011
≤ 90.40 %	22 (32%)	254	(0.320–1.044)		110	(0.285–0.963)	

^a^ Reference category for analysis.

As we previously reported, higher CA19‐9 levels, cfDNA concentration, cfDNA fragmentation, and higher *RAS* MAF load in cfDNA were associated with poor prognosis in mPDAC patients [20, 22]. Remarkably, *LAX1*, *RCAN3*, and *NR3C1* methylation levels also significantly stratified mPDAC patients according to OS and PFS. Specifically, *LAX1* methylation distinguished between low‐risk (419 days OS) and high‐risk (260 days OS) groups (*p =* 0.020; Figure 3A). Likewise, elevated *RCAN3* methylation levels were associated with the high‐risk group (395 days), whereas lower methylation levels were observed in the low‐risk group (194 days). Interestingly, elevated *NR3C1* methylation levels correlated with a more favorable prognosis, as reflected by a longer median OS of 391 days, compared to 254 days in patients with reduced *NR3C1* methylation levels (*p* = 0.032). Importantly, these associations remained significant when ROC curve‐derived cut‐off values were applied, indicating that the prognostic impact of these markers is robust to the method used for cut‐off selection (Figure S5). In addition, a significant difference in PFS was observed between high‐ and low‐risk groups stratified according to the levels of non‐epigenetic circulating biomarkers analyzed (Figure 3B).

To assess the independent prognostic value of the proposed methylation biomarkers, multivariable Cox proportional hazards models were constructed for OS and PFS (Table S2). Remarkably, *NR3C1*‐gene methylation emerged as an independent prognostic factor (*p* = 0.05), together with baseline CA19‐9 levels (*p* = 0.012) and treatment regimen (*p* = 0.001). For PFS, multivariable analysis identified methylation of *LAX1* gene (*p* = 0.044), cfDNA fragmentation (*p* = 0.006), *RAS* mutational status (*p* = 0.001), ECOG performance status (*p* = 0.003 and *p* = 0.013), and CA19‐9 levels (*p* = 0.001) as independent predictors of disease progression. Overall, these findings indicate that specific methylation markers retain independent prognostic value in multivariable models, with *NR3C1* methylation being independently associated with OS and *LAX1* methylation with PFS.

On the other hand, in those patients monitored using liquid biopsy following treatment initiation, plasma *NR3C1* early methylation changes were found to correlate with clinical outcomes and survival. Specifically, a significant negative correlation was found between circulating *NR3C1* methylation levels and survival (*r* = −0.337, *p* = 0.041; Figure S6). No significant correlations were observed for the remaining biomarkers, apart from cfDNA fragmentation, which also demonstrated a positive correlation with survival (*r* = 0.416, *p* = 0.008). These findings suggest that early changes in *NR3C1* methylation levels in plasma may serve as a biomarker of disease evolution in patients with mPDAC.

### 2.5 Baseline Circulating *NR3C1* Methylation Level Predicts FOLFIRINOX‐Based Regimen Outcome

**FIGURE 4:**
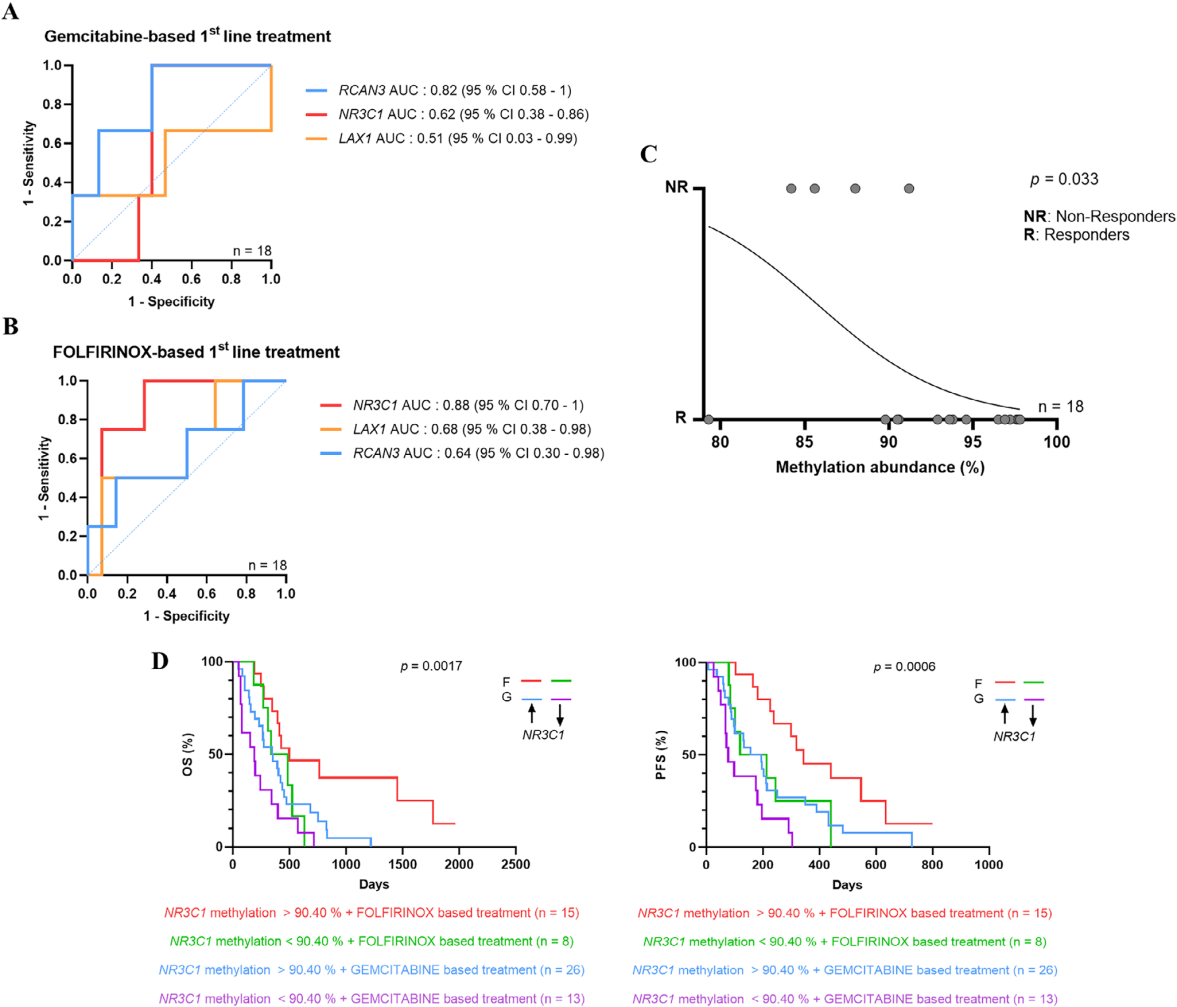
Basal circulating *NR3C1* methylation level predicts FOLFIRINOX‐based regimen outcome in mPDAC patients. ROC curve analysis showing the predictive value of basal methylation levels of *LAX1*, *RCAN3*, and *NR3C1* for disease progression at first follow‐up in mPDAC patients treated with (A) gemcitabine‐based or (B) FOLFIRINOX‐based first‐line regimens. (C) Simple logistic regression model evaluating the association between baseline *NR3C1* methylation and treatment outcome in FOLFIRINOX‐treated mPDAC patients. (D) Kaplan–Meier curve analysis of OS and PFS in mPDAC patients stratified by basal *NR3C1* methylation levels (90.4 % cut‐off) and first‐line treatment received (F: FOLFIRINOX; G: gemcitabine).

Next, we evaluated whether circulating methylation levels of *LAX1*, *RCAN3*, or *NR3C1* could predict treatment response. To this end, baseline methylation levels were compared with treatment outcomes (classified as responders or non‐responders) at the first follow‐up in two patient groups: 18 patients treated with FOLFIRINOX‐based therapy and 18 patients treated with gemcitabine‐based therapy. As shown by the ROC curve analyses (Figure 4), basal circulating methylation of none of the genes was found to be a reliable prognostic biomarker for first‐line gemcitabine‐based treatment (*RCAN3 p* = 0.086, *NR3C1 p* = 0.515; *LAX1 p* = 0.953; Figure 4A). In contrast, the basal circulating methylation level of *NR3C1* proved to be a useful predictive biomarker for treatment outcome in patients receiving FOLFIRINOX‐based therapy, with a sensitivity of 75%, specificity of 92.86%, and an area under the curve (AUC) of 0.88 (95% CI: 0.70–1; *p* = 0.026; Figure 4B). This predictive value was further supported by simple logistic regression analysis (Figure 4C), which confirmed the ability of circulating *NR3C1* methylation to stratify patients based on first‐line FOLFIRINOX‐based treatment response versus progression (non‐response; *p* = 0.033).

Subsequently, we evaluated the ability of circulating *NR3C1* methylation to stratify the full 69‐patient cohort in terms of OS and PFS, according to the treatment received: FOLFIRINOX or gemcitabine. As shown in Figure 4D, Kaplan–Meier analysis revealed that those patients with basal *NR3C1* methylation > 90.4 % that received first‐line FOLFIRINOX treatment exhibited better outcomes, with significantly improved OS (*p* = 0.0017) and PFS (*p* = 0.0006). These results reinforce the potential of *NR3C1* methylation as a valuable biomarker for predicting treatment response and improving patient stratification in mPDAC, particularly for those undergoing FOLFIRINOX‐based therapy.

### 2.6 Aberrant Methylation of the *NR3C1* Promoter Contributes to Its Transcriptional Downregulation in PDAC

To further investigate the role of *NR3C1* methylation in PDAC, DNA methylation profiles from The Cancer Genome Atlas (TCGA) were analyzed using the Shiny Methylation Analysis Resource Tool (SMART). The dataset included Illumina Human Methylation 450K data from 10 normal tissue samples and 184 PDAC tumor tissue samples. *NR3C1* promoter methylation was assessed by calculating the average β value across 17 CpGs within the CpG island located in the proximal promoter region. This analysis revealed significantly higher levels of *NR3C1* promoter methylation in PDAC tumors, compared to normal tissue (*p <* 0.0001; Figure S7A).

Next, we examined the relationship between *NR3C1* promoter methylation and gene expression in PDAC. Pearson correlation revealed a significant inverse correlation at 16 out of 17 CpGs sites within the proximal promoter CpG island, with correlation coefficients ranging from −0.45 to −0.13, *p <* 0.05 (Figure S7B,C). These results suggest that aberrant methylation of the *NR3C1* promoter contributes to its transcriptional downregulation in PDAC, with multiple CpG sites likely playing a critical role in silencing gene expression.

## 3 Discussion

Surgical resection is the only potentially curative option for PDAC, yet most patients present with unresectable disease at diagnosis [26]. Unresectable patients are typically managed with FOLFIRINOX‐ or gemcitabine‐based regimens [4]. Although FOLFIRINOX provides survival benefits, its substantial toxicity makes it unsuitable for many patients, and responses remain highly variable [9]. First‐line therapy selection relies mainly on the patient's ECOG performance status [10], prioritizing tolerability over therapeutic efficacy. Therefore, identifying predictive biomarkers to guide first‐line treatment selection and monitor disease progression remains a critical unmet need in mPDAC.

In this study, we assess the predictive value of liquid biopsy–derived epigenetic markers for treatment response in patients with metastatic PDAC. First, by using high‐throughput methylation array profiling and validation by ddPCR, we have demonstrated the aberrant methylation of three specific genes, *LAX1*, *RCAN3*, and *NR3C1*, in cfDNA from PDAC patients. These results underscore the potential value of these epigenetic alterations as candidate PDAC biomarkers that may contribute to the development of minimally invasive tools for disease detection and monitoring.

On the other hand, unlike these three targets, *KIAA1949* reached significance in the discovery array but did not show significant differences in ddPCR. This discrepancy may be due to several factors. There may be differences in CpG coverage between the array probe and the ddPCR assay, or it may reflect assay sensitivity and design, as in some cases, ddPCR may fail to detect subtle methylation differences reported by the array, particularly when the methylation change is small or the amount of input DNA is limited [27, 28].

The expression of *LAX1* has been shown to inversely correlate with DNA methylation levels [29], and several studies have reported that *LAX1* expression is negatively regulated by DNA hypermethylation [30, 31]. In this context, analyses from the TCGA Genomic Data Analysis Center identified the CpG site cg06958535 within *LAX1* as one of the CpG sites most strongly inversely correlated with gene expression in PDAC, showing a Spearman correlation of −0.81, supporting a methylation‐dependent regulation of *LAX1* expression [32]. Moreover, the expression of *LAX1* has been previously described as a positive prognostic biomarker in several cancer types. Specifically, *LAX1* expression is associated with favorable outcomes in ER‐/HER2 breast cancer [29], disease‐free survival when combined with other biomarkers in ovarian cancer [33], and recurrence‐free survival in colorectal cancer [34]. These findings support our observations in mPDAC patients, where higher *LAX1* methylation levels were associated with shorter OS and PFS. Furthermore, *LAX1* methylation levels significantly correlated with previously reported negative prognostic cfDNA‐based markers in metastatic PDAC, such as total cfDNA concentration, cfDNA fragmentation, and *RAS* MAF [20, 35], reinforcing the clinical relevance of *LAX1* methylation status in mPDAC. Notably, while *LAX1* expression has also been linked to increased sensitivity to platinum‐based chemotherapy in ovarian cancer [33] and to FOLFOX in colorectal cancer [34], such associations have not yet been demonstrated in the context of PDAC.

*RCAN3* (RCAN Family Member 3) is located on chromosome 1 (1p36.11) and plays a role in calcium signaling through its function as a regulator of calcineurin [36]. Previous studies have demonstrated that *RCAN3* expression is negatively regulated by DNA methylation [37]. Furthermore, *RCAN3* has been characterized as a tumor suppressor gene, with reported roles in inhibiting tumor growth and angiogenesis in a human breast cancer model [38]. These findings are consistent with our current results from array and validation analyses, where increased methylation levels of *RCAN3* were observed in plasma samples from mPDAC patients. Kaplan–Meier survival analyses further revealed that patients classified in the high‐risk group for *RCAN3* methylation exhibited significantly lower OS and PFS. This observation agrees with findings by Wang et al., who reported that higher *RCAN3* expression was associated with improved prognosis in patients with pheochromocytoma and paraganglioma, suggesting a potential context‐dependent role of *RCAN3* in tumor progression and patient outcome [39].

*NR3C1* is located on chromosome 5 (5q31.3) and encodes the glucocorticoid receptor (GR) [40, 41, 42]. *NR3C1* is broadly expressed across most cell types, with particularly high expression levels in pancreatic islets [43, 44]. As a transcription factor, *GR* modulates various molecular pathways by binding to glucocorticoid response elements, though its downstream effects can vary depending on cell type and tissue context, and it is differentially expressed in healthy and tumor tissues [43, 45]. *NR3C1* is known to regulate a variety of signaling cascades, including those associated with inflammatory responses like NF‐κB and cell survival through SGK1, as well as broader biological processes, including differentiation, energy metabolism, immunity, neuroendocrine integration, and cell cycle regulation [41, 46, 47].

*NR3C1* expression has been shown to be reduced in several cancer types, including lung, bladder, prostate, and cervical cancers, compared to corresponding normal tissues [45]. Consistent with previous reports [48, 49], our analysis of TCGA data indicates that increased methylation of the *NR3C1* promoter is associated with its reduced transcription in PDAC. Besides, our array and validation analyses identified significantly higher circulating *NR3C1* methylation levels in metastatic PDAC patients, compared to healthy controls, supporting a potential role of *NR3C1* silencing in advanced disease. However, our survival analysis revealed an association of elevated circulating *NR3C1* methylation levels and better outcome in metastatic PDAC. Moreover, low *NR3C1* methylation levels were consistently detected in samples characterized by higher values of CA19‐9, *RAS* MAF, and cfDNA concentration and fragmentation. Deng et al. reported in 2021 that higher *NR3C1* expression in PDAC is associated with poorer patient survival [50]. Notably, our results align with these previous findings, suggesting that elevated *NR3C1* methylation, and consequently reduced expression, may be associated with improved outcomes in metastatic PDAC patients treated with FOLFIRINOX‐based chemotherapy regimens.

Furthermore, although the role of *NR3C1* as a transcription factor is well established, recent evidence has uncovered an additional function in mediating chemotherapy resistance. In gastric cancer, *NR3C1* has been recently identified as a component of a transcriptional complex contributing to resistance to 5‐Fluorouracil [51], which is a key component of the FOLFIRINOX regimen, by promoting the transcription of downstream resistance‐associated genes [52]. In fact, *NR3C1* has been shown to directly regulate *ABCC1*, which encodes the ABC transporter MRP1 [51], a membrane protein involved in the efflux of multiple chemotherapeutic agents, including FOLFIRINOX components such as irinotecan and leucovorin, thereby limiting their intracellular accumulation [53]. This *NR3C1–ABCC1* axis provides a plausible molecular mechanism through which altered *NR3C1* regulation may modulate FOLFIRINOX sensitivity. In the context of PDAC, response to FOLFIRINOX has been shown to differ by molecular subtype. Lansbergen et al. demonstrated that the classical and basal‐like subtypes, as defined by Moffitt et al. in 2015, remain the most reliable molecular classification for metastatic PDAC, with the basal‐like subtype exhibiting greater aggressiveness and reduced sensitivity to FOLFIRINOX treatment [9, 13, 54]. Notably, *NR3C1* expression in the basal‐like subtype is nearly four times higher than in the classical subtype [13]. Consistent with this, our study demonstrates that high circulating *NR3C1* methylation levels, which may be related to low *NR3C1* expression in tumor, is associated with better outcomes with FOLFIRINOX‐based therapies. Collectively, these results suggest that circulating *NR3C1* methylation may be subtype‐dependent and could be associated with different treatment outcomes in PDAC.

Recent studies have shown that *AGRN‐driven* epithelial‐mesenchymal transition (EMT) may constitute a molecular program responsible for aggressive and treatment‐refractory states in gastrointestinal cancers [55] and that the innovative nanodelivery of gemcitabine and deferasirox using M1 macrophage‐derived exosomes has demonstrated the ability to overcome chemotherapy resistance in pancreatic cancer [56]. Together with our findings, these studies support the need for reliable biomarkers capable of capturing resistance‐associated tumor states. In this context, circulating epigenetic markers, such as *NR3C1* methylation, may provide a minimally invasive readout of underlying tumor biology and help support treatment stratification.

In summary, our study underscores the potential of circulating epigenetic biomarkers, specifically *LAX1*, *RCAN3*, and *NR3C1* methylation levels, as promising tools for monitoring disease progression and predicting treatment response in metastatic PDAC. These biomarkers offer a minimally invasive approach to inform therapeutic decisions, with circulating *NR3C1* methylation showing potential in predicting response to FOLFIRINOX‐based chemotherapy. Future studies are essential to elucidate the underlying mechanisms of these associations and to further refine the clinical application of these biomarkers in personalized PDAC management.

This study has certain limitations that should be considered. First, the genome‐wide methylation analysis was performed in a small discovery cohort, which increases the risk of both false‐positive and false‐negative results. However, the subsequent targeted validation by ddPCR in a larger sample set mitigates this concern and supports the robustness of the main findings. Besides, the imbalance in sample size between patients and controls may limit statistical power for specificity assessment and could influence false‐positive rates, so further studies with larger, more balanced cohorts are granted. Second, although the number of healthy controls was limited and the median age differed from that of patients, additional correlation and regression analyses demonstrated no significant association between age and methylation levels for any of the validated genes, suggesting minimal confounding by age. Third, subgroup analyses were limited by small patient numbers per treatment arm, making the predictive performance of *NR3C1* preliminary and requiring validation in larger, independent, and prospective cohorts. On the other hand, future multicenter studies are warranted to confirm the reproducibility and clinical applicability of these biomarkers, including the methylation threshold determined for *NR3C1*, across diverse populations and analytical settings. Finally, inherent technical and standardization challenges associated with cfDNA methylation analysis and liquid biopsy—such as pre‐analytical variability, assay sensitivity, and inter‐platform comparability—remain important considerations for clinical translation.

Despite these limitations, the overall consistency of our findings across methods and datasets provides strong preliminary evidence supporting the biological significance and potential clinical relevance of the identified methylation markers, laying the groundwork for future multicenter prospective studies aimed at translating these biomarkers into clinical practice.

## 4 Conclusion

In this study, we demonstrated the prognostic value of *LAX1*, *NR3C1*, and *RCAN3* methylation in patients with mPDAC, reinforcing the potential of liquid biopsy‐based epigenetic biomarkers in clinical practice to improve mPDAC management. Notably, we identified diagnostic *NR3C1* methylation as a predictive biomarker for first‐line treatment response, supporting its utility in guiding clinical decision‐making. Future studies will aim to elucidate the role of *NR3C1* methylation in FOLFIRINOX resistance, potentially revealing new therapeutic targets.

## 5 Methods

### 5.1 Patients and Samples

For this study, 69 patients were prospectively enrolled at the Reina Sofía University Hospital (HURS) in Córdoba (Spain), between May 2017 and September 2023. Eligibility criteria included adults (≥18 years) with histologically verified mPDAC, no history of chemotherapy or radiotherapy, and provision of written informed consent. The research adhered to the principles of the Declaration of Helsinki and obtained approval from the Córdoba Ethics Committee (Metilpancreas2 protocol, approved on November 29, 2022, Act 343, reference 5507). Table 1 provides a summary of the baseline characteristics of the patients enrolled in the study.

At diagnosis, blood samples were collected from 69 patients. During follow‐up, an additional 62 samples were obtained from 39 of these patients, aligned with CT‐based assessments of disease progression and continued until progression or death. In total, 131 patient samples were collected for analysis. The healthy cohort consisted of six women and five men, aged between 22 and 61 years, with a median age of 41 years.

Plasma was obtained by centrifuging blood samples at 1600 x *g* for 10 min at room temperature (RT) and removing any cellular debris by further centrifugation at 6000 x *g* for 10 min at RT. Plasma samples were aliquoted and cryopreserved at −80°C prior to analysis.

CA19‐9 levels (U/mL) were quantified in the hospital Clinical Laboratory using a standard radioimmunoassay.

### 5.2 Plasma cfDNA Isolation and cfDNA Fragmentation Analysis

cfDNA was purified from 3 mL of plasma using the QIAamp Circulating Nucleic Acid Kit in combination with the QIAvac 24 Plus vacuum system (Qiagen). The extracted cfDNA was quantified with a Quantus fluorometer (Promega), and the samples were stored at −80°C to maintain their integrity.

cfDNA fragmentation was assessed on the Agilent 2200 TapeStation system (Agilent) using the High Sensitivity D1000 ScreenTape Assay. cfDNA fragmentation was quantified as the fraction of short fragments compared with the total cfDNA content.

### 5.3 *RAS* MAF Assessment

Mutation analysis was performed either by the OncoBEAM *RAS* assay (Sysmex Inostics) or by the Plasma‐SeqSensei (PSS, Sysmex Inostics). Briefly, the OncoBEAM *RAS* Assay begins with a conventional PCR to amplify the target locus, which includes seven amplicons covering codons 12, 13, 59, 61, 117, and 146 and detects 34 mutations in the *KRAS/NRAS* genes. For each codon, a digital PCR was conducted, and the cfDNA was hybridized with fluorescent probes to measure mutant and wild‐type *KRAS/NRAS* molecules using flow cytometry. This method enables the accurate detection of mutant allele frequencies below 0.1 % in cfDNA [57]. Plasma‐SeqSensei is a next‐generation sequencing assay, which detects *BRAF*, *KRAS*, *NRAS*, and *PIK3CA* mutations in cfDNA [58]. The high sensitivity of this method relies in the labeling of DNA fragments with unique molecular identifiers (UIDs) during the first amplification step. Then, in the second amplification, each member of the same family of UIDs is identified with specific barcodes. Sequencing was performed on MiSeq (Illumina), and the Plasma SeqSensei software was used for data acquisition and analysis, reporting the proportion of mutated molecules relative to the total cfDNA (MAF).

### 5.4 Infinium MethylationEPIC BeadChip Array

The genome‐wide DNA methylation was quantified using the Infinium MethylationEPIC BeadChip Array via Diagenode S.A. (Belgium). This array interrogates 866,895 single‐nucleotide methylation sites, covering about 99% of annotated RefSeq genes.

This array encompasses 866,895 single‐nucleotide methylation sites and cover 99% of the annotated reference sequence (RefSeq) genes.

The discovery set included cfDNA samples from six mPDAC patients and two healthy individuals. Extracted cfDNA was measured with the Quantus fluorometer (Promega). A total of 25 ng was deaminated with the EZ‐96 DNA Methylation Kit (Zymo Research) according to Illumina's protocol. Bisulfite conversion efficiency was evaluated by qPCR, targeting a methylated region of DNAJC15 and the GNAS locus, which were used for quality control.

Comparative analysis of data was conducted using the Bioconductor R package Chip Analysis Methylation Pipeline (ChAMP) [59]. After normalization of the beta values with BMIQ [60], potential differentially methylated probes were identified using a Benjamini–Hochberg adjusted *p*‐value < 0.05, with no threshold applied to the methylation differences. The GRCh37/hg19 genome, obtained from the UCSC Genome Browser, was used as a reference.

### 5.5 cfDNA Sodium Bisulfite Conversion

A maximum of 25 µL of cfDNA sample was subjected to sodium bisulfite treatment using the EZ DNA Methylation‐Lightning kit (Zymo Research), following the manufacturer's protocol. Human HCT116 DKO methylated and unmethylated DNAs were used as control samples to assess cfDNA bisulfite conversion efficiency in each treatment intended for ddPCR.

### 5.6 ddPCR

Prior to ddPCR analysis, target sequences were subjected to a pre‐amplification step following established protocols [22]. Briefly, a 10 µL reaction mixture was prepared containing 1 µL of bisulfite converted cfDNA, 2 × ddPCR Supermix (without dUTP, Bio‐Rad) and 900 nM of each primer. PCR conditions were 95°C for 10 min, followed by 10 cycles of 94°C for 30 s and 58°C for 1 min and a final incubation at 98°C for 10 min. Finally, all pre‐amplified PCR products were diluted 1:10.

ddPCR was conducted using the QX200 system (Bio‐Rad). For each assay, 2 µL of the diluted pre‐amplification product was mixed with ddPCR Supermix, 900 nM of primers, and 250 nM of the corresponding probes (FAM or SUN fluorophore‐labelled (Integrated DNA Technologies, Inc.), in a final volume of 20 µL. To minimize background noise and enhance assay sensitivity, all probes were quenched using a 30 Iowa Black dark quencher (IABkFQ) and an internal ZEN quencher. When the positive droplets for methylated and unmethylated probes did not correctly separate from background, Affinity Plus probes were used. These probes enhance the discrimination of thermodynamically similar samples by incorporating up to six blocked nucleic acid monomers. The locked nucleic acids confer greater structural stability, which increases the hybridization melting temperature. The primer and probe sequences used are specified in Table S3. The ddPCR conditions were as follows: 95°C for 10 min, followed by 40 cycles of 94°C for 30 s, 58°C for 1 min, and a final step at 98°C for 10 min. The PCR products were analyzed using the QX200 droplet reader, which quantified the total number of droplets, and determined the counts of positive and negative droplets for each fluorophore. QuantaSoft Analysis Pro 1.0.596 program was employed to calculate the relative methylation abundance for each sample, defined as [number of methylated copies (FAM) / (number of methylated copies (FAM) + unmethylated copies (SUN))] * 100.

### 5.7 TCGA Database and Statistical Analysis

TCGA‐PAAD project data were analyzed using the SMART website (http://www.bioinfo‐zs.com/smartapp/) [61] to evaluate differential methylation levels in *NR3C1* CpG island between normal and PDAC tissue samples, as well as to assess the correlation with gene expression.

SPSS Statistics version 26.0.0, GraphPad Prism 9.0 software and R version 4.4.2 were used for statistical analysis of the data. To account for potential age‐related effects on methylation between healthy and patient groups, a multiple linear regression model was applied for each validated target, including group and age as independent variables (*methylation = group + age*). The R2 Genomics Analysis and Visualization Platform (https://r2.amc.nl) was used to establish the cut‐off values of *NR3C1*, *RCAN3*, and *LAX1* methylation levels, MAF, CA19‐9 levels, cfDNA concentration and cfDNA fragmentation. As R2 provides a data‐driven approach optimized for group separation but not explicitly designed for prognostic threshold definition, receiver operating characteristic (ROC) curve analyses were subsequently conducted to derive outcome‐oriented cut‐off values for OS and PFS. OS and progression‐free survival were defined as the time from diagnosis, based on tissue biopsy, to death from any cause or to the first CT‐progression, respectively. The statistical significance in the digital droplet PCR validation and the association analysis was determined using the non‐parametric Mann–Whitney test or the parametric unpaired *t*‐test. Correlations were assessed using the Pearson correlation coefficient. To control for multiple testing, *p*‐values from correlation analyses between circulating gene methylation levels and molecular biomarkers were adjusted separately for each gene using the Benjamini–Hochberg false discovery rate (FDR) method. Correlations with an adjusted *q*‐value ≤ 0.05 were considered statistically significant. Survival rates were estimated using Kaplan–Meier curves, and log‐rank test was employed to identify prognostic variables. Patient stratification for logistic simple regression and ROC curve analysis was based on response or non‐response to first‐line treatment. Statistical significance was defined as *p* < 0.05 for all analyses.

## Author Contributions

P.C‐R., M.V.G‐O., A.R‐A., and E.A. conceived, designed, and supervised the study. M.T.C. and E.A. provided resources. P.C‐R., M.V.G‐O., M.T‐F., and E.C‐P. performed experiments and conducted data analysis. N.H‐C. was responsible for the bioinformatic analyses. E.A. and M.V.G‐O. acquired funding. P.C‐R., M.V.G‐O., and A.R‐A. wrote the manuscript. All authors reviewed and approved the final version of the manuscript.

## Funding

This research was supported by grants to M.V.G‐O. and E.A. from Consejería de Universidad, Investigación e Innovación, Junta de Andalucía (ProyExcel_00734), Instituto de Salud Carlos III (PI25/00503) co‐funded by the European Union and a grant to M.V.G‐O. from Beca Carmen Delgado/Miguel Pérez‐Mateo 2024. M.V.G‐O. and A.R‐A. are funded with a researcher contract through the Program “Nicolás Monardes” from Junta de Andalucía.

## Conflicts of Interest

The authors declare no conflicts of interest.

## Ethics Statement

The research followed the ethical principles outlined in the World Medical Association Declaration of Helsinki and received approval from the Ethics Committee of Córdoba. (Metilpancreas2 protocol, approved on November 29, 2022, Act 343, reference 5507).

## Supporting information




**Figure S1**: Comparative analysis of CpG methylation levels of *KIAA1949* gene in plasma samples from healthy individuals and mPDAC patients.
**Figure S2**: Residuals versus fitted values plots from multiple linear regression analyses evaluating the potential confounding effect of age on methylation levels for *LAX1*, *RCAN3*, and *NR3C1* genes (model: methylation = group + age). Residuals were randomly distributed around zero, indicating no systematic bias and supporting that age does not significantly influence the observed group differences. Fitted values (X‐axis): predicted methylation levels estimated by the linear model (adjusted for group and age). Residuals (Y‐axis): the difference between the observed and predicted methylation values. *p*‐values correspond to the regression coefficient for age, indicating the lack of a significant association between age and methylation levels (*p* > 0.05).
**Figure S3**: Correlation between basal circulating *LAX1*, *RCAN3*, and *NR3C1* methylation levels with other circulating tumor biomarkers in mPDAC. *LAX1, RCAN3*, and *NR3C1* methylation levels in plasma at diagnosis according to CA19‐9 levels, cfDNA concentration, cfDNA fragmentation and *RAS* MAF. Pearson's correlation *p*‐values and Benjamini–Hochberg false discovery rate (FDR)‐adjusted *q*‐values are shown.
**Figure S4**: (A) cfDNA concentration and (B) cfDNA fragmentation in healthy individuals, compared with metastatic (stage IV) pancreatic cancer patients.
**Figure S5**: Kaplan–Meier survival analyses of mPDAC patients stratified according to ROC curve‐derived cut‐off values for *LAX1*, *RCAN3*, and *NR3C1* methylation markers. Overall survival (OS) and progression‐free survival (PFS) were compared between high‐ and low‐methylation groups using the log‐rank test. Results are consistent with those obtained using R2‐defined cut‐off values.
**Figure S6**: Correlation between early changes of circulating biomarkers and progression‐free survival. Graphs show the slope from diagnosis to first follow‐up after treatment initiation of *LAX1* methylation, *RCAN3* methylation, *NR3C1* methylation, CA19‐9 levels, *RAS* MAF, cfDNA concentration and cfDNA fragmentation.
**Figure S7**: Aberrant methylation of the *NR3C1* promoter contributes to its transcriptional downregulation in PDAC. (A) Average methylation of 17 CpGs sites within the proximal promoter CpG island in normal tissue and PDAC tumor tissue samples. (B) Pearson correlation coefficient for each CpG position. (C) Correlation between global proximal promoter CpG island methylation and *NR3C1* mRNA levels. All data were from TCGA database.
**Table S1**: Methylation levels of 9 CpG positions (adjusted *p*‐value ≤ 0.05; Δβ ≥ 0.20) with negative and positive controls of methylation. ± SD indicate Poisson's error (95% CI).
**Table S2**: Multivariable Cox regression analysis for OS and progression‐free survival according to basal circulating biomarkers in mPDAC patients.
**Table S3**: Primers and probes for methylation analysis of different genes in cfDNA.

## Data Availability

The MethylationEPIC BeadChip Array data have been deposited in the National Center for Biotechnology Information (NCBI) Gene Expression Omnibus (GEO) repository (https://www.ncbi.nlm.nih.gov/geo/) under accession number GSE316251.

## References

[1] H. Wei and H. Ren , “Precision Treatment of Pancreatic Ductal Adenocarcinoma,” Cancer Letters 585 (2024): 216636. 38278471 10.1016/j.canlet.2024.216636

[2] C. Bosetti , V. Rosato , D. Li , et al., “Diabetes, Antidiabetic Medications, and Pancreatic Cancer Risk: An Analysis From the International Pancreatic Cancer Case‐Control Consortium,” Annals of Oncology 25, no. 10 (2014): 2065–2072. 25057164 10.1093/annonc/mdu276 PMC4176453

[3] J. D. Mizrahi , R. Surana , J. W. Valle , and R. T. Shroff , “Pancreatic Cancer,” Lancet 395, no. 10242 (2020): 2008–2020. 32593337 10.1016/S0140-6736(20)30974-0

[4] W. Park , A. Chawla , and E. M. O'Reilly , “Pancreatic Cancer: A Review,” Jama 326, no. 9 (2021): 851–862. 34547082 10.1001/jama.2021.13027 PMC9363152

[5] J. X. Hu , C. F. Zhao , W. B. Chen , et al., “Pancreatic Cancer: A Review of Epidemiology, Trend, and Risk Factors,” World Journal of Gastroenterology 27, no. 27 (2021): 4298–4321. 34366606 10.3748/wjg.v27.i27.4298 PMC8316912

[6] I. Garajová , M. Peroni , F. Gelsomino , and F. Leonardi , “A Simple Overview of Pancreatic Cancer Treatment for Clinical Oncologists,” Current Oncology 30, no. 11 (2023): 9587–9601. 37999114 10.3390/curroncol30110694 PMC10669959

[7] X. Y. He and Y. Z. Yuan , “Advances in Pancreatic Cancer Research: Moving Towards Early Detection,” World Journal of Gastroenterology 20, no. 32 (2014): 11241–11248. 25170208 10.3748/wjg.v20.i32.11241 PMC4145762

[8] A. Carrato , A. Falcone , M. Ducreux , et al., “A Systematic Review of the Burden of Pancreatic Cancer in Europe: Real‐World Impact on Survival, Quality of Life and Costs,” Journal of Gastrointestinal Cancer 46, no. 3 (2015): 201–211. 25972062 10.1007/s12029-015-9724-1 PMC4519613

[9] M. F. Lansbergen , M. P. G. Dings , P. Manoukian , et al., “Transcriptome‐Based Classification to Predict FOLFIRINOX Response in a Real‐World Metastatic Pancreatic Cancer Cohort,” Translational Research 273 (2024): 137–147. 39154856 10.1016/j.trsl.2024.08.002

[10] T. Conroy , F. Desseigne , M. Ychou , et al., “FOLFIRINOX Versus Gemcitabine for Metastatic Pancreatic Cancer,” New England Journal of Medicine 364, no. 19 (2011): 1817–1825. 21561347 10.1056/NEJMoa1011923

[11] E. N. Pijnappel , W. P. M. Dijksterhuis , and G. L. G. der , “First‐ and Second‐Line Palliative Systemic Treatment Outcomes in a Real‐World Metastatic Pancreatic Cancer Cohort,” Journal of the National Comprehensive Cancer Network 20, no. 5 (2021): 443–450. 34450595 10.6004/jnccn.2021.7028

[12] M. Taherian , H. Wang , and H. Wang , “Pancreatic Ductal Adenocarcinoma: Molecular Pathology and Predictive Biomarkers,” Cells 11, no. 19 (2022): 3068. 36231030 10.3390/cells11193068 PMC9563270

[13] R. A. Moffitt , R. Marayati , E. L. Flate , et al., “Virtual Microdissection Identifies Distinct Tumor‐ and Stroma‐Specific Subtypes of Pancreatic Ductal Adenocarcinoma,” Nature Genetics 47, no. 10 (2015): 1168–1178. 26343385 10.1038/ng.3398 PMC4912058

[14] E. A. Collisson , P. Bailey , and D. K. Chang , “Biankin AV. Molecular Subtypes of Pancreatic Cancer,” Nature Reviews Gastroenterology & Hepatology 16, no. 4 (2019): 207–220. 30718832 10.1038/s41575-019-0109-y

[15] B. Zhao , B. Zhao , and F. Chen , “Diagnostic Value of Serum Carbohydrate Antigen 19‐9 in Pancreatic Cancer: A Systematic Review and Meta‐Analysis,” European Journal of Gastroenterology & Hepatology 34, no. 9 (2022): 891. 35913776 10.1097/MEG.0000000000002415

[16] E. D. Saad , M. C. Machado , D. Wajsbrot , et al., “Pretreatment CA 19‐9 Level as a Prognostic Factor in Patients With Advanced Pancreatic Cancer Treated With Gemcitabine,” International Journal of Gastrointestinal Cancer 32, no. 1 (2002): 35–41. 12630768 10.1385/IJGC:32:1:35

[17] T. M. Bauer , B. F. El‐Rayes , X. Li , et al., “Carbohydrate Antigen 19‐9 Is a Prognostic and Predictive Biomarker in Patients With Advanced Pancreatic Cancer Who Receive Gemcitabine‐Containing Chemotherapy: A Pooled Analysis of 6 Prospective Trials,” Cancer 119, no. 2 (2013): 285–292. 22786786 10.1002/cncr.27734 PMC4261189

[18] A. J. Bronkhorst , V. Ungerer , and S. Holdenrieder , “The Emerging Role of Cell‐Free DNA as a Molecular Marker for Cancer Management,” Biomolecular Detection and Quantification 17 (2019): 100087. 30923679 10.1016/j.bdq.2019.100087 PMC6425120

[19] M. Ilié and P. Hofman , “Pros: Can Tissue Biopsy be Replaced by Liquid Biopsy?” Translational Lung Cancer Research 5, no. 4 (2016): 420–423. 27655109 10.21037/tlcr.2016.08.06 PMC5009092

[20] M. Toledano‐Fonseca , M. T. Cano , E. Inga , et al., “Circulating Cell‐Free DNA‐Based Liquid Biopsy Markers for the Non‐Invasive Prognosis and Monitoring of Metastatic Pancreatic Cancer,” Cancers 12, no. 7 (2020): E1754. 10.3390/cancers12071754 PMC7409337 32630266

[21] M. V. García‐Ortiz , P. Cano‐Ramírez , M. Toledano‐Fonseca , et al., “Diagnosing and Monitoring Pancreatic Cancer Through Cell‐Free DNA Methylation: Progress and Prospects,” Biomarker Research 11, no. 1 (2023): 88. 37798621 10.1186/s40364-023-00528-y PMC10552233

[22] M. V. García‐Ortiz , P. Cano‐Ramírez , M. Toledano‐Fonseca , et al., “Circulating NPTX2 Methylation as a Non‐Invasive Biomarker for Prognosis and Monitoring of Metastatic Pancreatic Cancer,” Clinical Epigenetics 15 (2023): 118. 37481552 10.1186/s13148-023-01535-4 PMC10362605

[23] Q. Guo and W. Qin , “DKK3 Blocked Translocation of β‐Catenin/EMT Induced by Hypoxia and Improved Gemcitabine Therapeutic Effect in Pancreatic Cancer Bxpc‐3 Cell,” Journal of Cellular and Molecular Medicine 19, no. 12 (2015): 2832–2841. 26395974 10.1111/jcmm.12675 PMC4687707

[24] P. C. Mahon , P. Baril , V. Bhakta , et al., “S100A4 Contributes to the Suppression of BNIP3 Expression, Chemoresistance, and Inhibition of Apoptosis in Pancreatic Cancer,” Cancer Research 67, no. 14 (2007): 6786–6795. 17638890 10.1158/0008-5472.CAN-07-0440

[25] C. Zhang , S. Ou , Y. Zhou , et al., “m6A Methyltransferase METTL14‐Mediated Upregulation of Cytidine Deaminase Promoting Gemcitabine Resistance in Pancreatic Cancer,” Frontiers in Oncology 11 (2021): 696371. 34458141 10.3389/fonc.2021.696371 PMC8385558

[26] M. E. Gómez García , F. Carbonell Castelló , A. Alberola Soler , et al., “‘Second‐look’ en Adenocarcinoma de Páncreas Inicialmente Irresecable tras Quimioterapia Neoadyuvante,” Cirugía Española 91, no. 10 (2013): 683–685. 23498348 10.1016/j.ciresp.2012.05.027

[27] J. Roessler , O. Ammerpohl , J. Gutwein , et al., “Quantitative Cross‐Validation and Content Analysis of the 450k DNA Methylation Array From Illumina, Inc,” BMC Research Res Notes 5, no. 1 (2012): 210. 10.1186/1756-0500-5-210 PMC3420245 22546179

[28] L. Van Wesenbeeck , L. Janssens , H. Meeuws , et al., “Droplet Digital PCR Is an Accurate Method to Assess Methylation Status on FFPE Samples,” Epigenetics 13, no. 3 (2018): 207–213. 29527977 10.1080/15592294.2018.1448679 PMC5997148

[29] B. Győrffy , G. Bottai , T. Fleischer , et al., “Aberrant DNA Methylation Impacts Gene Expression and Prognosis in Breast Cancer Subtypes,” International Journal of Cancer 138, no. 1 (2016): 87–97. 26174627 10.1002/ijc.29684

[30] J. Liu , J. Chen , S. Ehrlich , et al., “Methylation Patterns in Whole Blood Correlate With Symptoms in Schizophrenia Patients,” Schizophrenia Bulletin 40, no. 4 (2014): 769–776. 23734059 10.1093/schbul/sbt080 PMC4059425

[31] H. Luo , H. Zhu , D. Bao , et al., “Genome‐Wide DNA Methylation and mRNA Transcription Analysis Revealed Aberrant Gene Regulation Pathways in Patients With Dermatomyositis and Polymyositis,” Chinese Medical Journal 138, no. 1 (2025): 120. 39497373 10.1097/CM9.0000000000003337 PMC11717519

[32] Broad Institute TCGA Genome Data Analysis Center . Correlation Between mRNA Expression and DNA Methylation . (Broad Institute of MIT and Harvard, 2016).

[33] F. Yin , S. Yi , L. Wei , et al., “Microarray‐Based Identification of Genes Associated With Prognosis and Drug Resistance in Ovarian Cancer,” Journal of Cellular Biochemistry 120, no. 4 (2019): 6057–6070. 30335894 10.1002/jcb.27892

[34] H. Huang , C. Wu , A. Colaprico , et al., “Discovery of Oncogenic Mediator Genes in Rectal Cancer Chemotherapy Response Using Gene Expression Data From Matched Tumor and Patient‐Derived Organoid,” preprint, medRxiv, January 30, 2024.

[35] M. Lapin , S. Oltedal , K. Tjensvoll , et al., “Fragment Size and Level of Cell‐Free DNA Provide Prognostic Information in Patients With Advanced Pancreatic Cancer,” Journal of translational medicine 16 (2018): 300. 30400802 10.1186/s12967-018-1677-2 PMC6218961

[36] Y. Liang , W. Diao , X. Yang , et al., “Regulator of Calcineurin 3 as a Novel Predictor of Diagnosis and Prognosis in Pan‐Cancer,” Croatian Medical Journal 65, no. 4 (2024): 356–372. 39219199 10.3325/cmj.2024.65.356 PMC11399725

[37] E. Serrano‐Candelas , D. Farré , Á. Aranguren‐Ibáñez , et al., “The Vertebrate RCAN Gene Family: Novel Insights Into Evolution, Structure and Regulation,” PLoS ONE 9, no. 1 (2014): e85539. 24465593 10.1371/journal.pone.0085539 PMC3896409

[38] S. Martínez‐Høyer , S. Solé‐Sánchez , F. Aguado , et al., “A Novel Role for an RCAN3‐derived Peptide as a Tumor Suppressor in Breast Cancer,” Carcinogenesis 36, no. 7 (2015): 792–799. 25916653 10.1093/carcin/bgv056

[39] Z. Wang , Y. Li , Y. Zhong , Y. Wang , and M. Peng , “Comprehensive Analysis of Aberrantly Expressed Competitive Endogenous RNA Network and Identification of Prognostic Biomarkers in Pheochromocytoma and Paraganglioma,” OncoTargets and Therapy 13 (2020): 11377–11395. 33192072 10.2147/OTT.S271417 PMC7654541

[40] L. Li , W. Xing , L. Jiang , D. Chen , and G. Zhang , “NR3C1 Overexpression Regulates the Expression and Alternative Splicing of Inflammation‐associated Genes Involved in PTSD,” Gene 859 (2023): 147199. 36657650 10.1016/j.gene.2023.147199

[41] G. E. Lind , K. Kleivi , G. I. Meling , et al., “ADAMTS1, CRABP1, and NR3C1 Identified as Epigenetically Deregulated Genes in Colorectal Tumorigenesis,” Cellular Oncology 28, no. 5‐6 (2006): 259–272. 17167179 10.1155/2006/949506 PMC4618201

[42] L. M. Noureddine , O. Trédan , N. Hussein , et al., “Glucocorticoid Receptor: A Multifaceted Actor in Breast Cancer,” International Journal of Molecular Sciences 22, no. 9 (2021): 4446. 33923160 10.3390/ijms22094446 PMC8123001

[43] S. Khadka , S. R. Druffner , B. C. Duncan , and J. T. Busada , “Glucocorticoid Regulation of Cancer Development and Progression,” Frontiers in Endocrinology 14 (2023): 1161768. 37143725 10.3389/fendo.2023.1161768 PMC10151568

[44] T. Wu and Y. Shao , “NR3C1/Glucocorticoid Receptor Activation Promotes Pancreatic β‐Cell Autophagy Overload in Response to Glucolipotoxicity,” Autophagy 19, no. 9 (2023): 2538–2557. 37039556 10.1080/15548627.2023.2200625 PMC10392762

[45] N. Bakour , F. Moriarty , G. Moore , et al., “Prognostic Significance of Glucocorticoid Receptor Expression in Cancer: A Systematic Review and Meta‐Analysis,” Cancers 13, no. 7 (2021): 1649. 33916028 10.3390/cancers13071649 PMC8037088

[46] L. C. Matthews , A. A. Berry , D. J. Morgan , et al., “Glucocorticoid Receptor Regulates Accurate Chromosome Segregation and Is Associated With Malignancy,” PNAS 112, no. 17 (2015): 5479–5484. 25847991 10.1073/pnas.1411356112 PMC4418855

[47] S. Prekovic , K. Schuurman , I. Mayayo‐Peralta , et al., “Glucocorticoid Receptor Triggers a Reversible Drug‐tolerant Dormancy State With Acquired Therapeutic Vulnerabilities in Lung Cancer,” Nature Communications 12 (2021): 4360. 10.1038/s41467-021-24537-3 PMC8285479 34272384

[48] E. Ayroldi , L. Cannarile , D. V. Delfino , and C. Riccardi , “A Dual Role for Glucocorticoid‐Induced Leucine Zipper in Glucocorticoid Function: Tumor Growth Promotion or Suppression?,” Cell Death & Disease 9, no. 5 (2018): 1–12. 29695779 10.1038/s41419-018-0558-1 PMC5916931

[49] H. Snider , B. Villavarajan , Y. Peng , et al., “Region‐Specific Glucocorticoid Receptor Promoter Methylation Has Both Positive and Negative Prognostic Value in Patients With Estrogen Receptor‐Positive Breast Cancer,” Clinical Epigenetics 11, no. 1 (2019): 155. 31675993 10.1186/s13148-019-0750-x PMC6825343

[50] Y. Deng , X. Xia , Y. Zhao , et al., “Glucocorticoid Receptor Regulates PD‐L1 and MHC‐I in Pancreatic Cancer Cells to Promote Immune Evasion and Immunotherapy Resistance,” Nature Communications 12, no. 1 (2021): 7041. 10.1038/s41467-021-27349-7 PMC8649069 34873175

[51] J. Yu , M. Chen , Q. Sang , et al., “Super‐Enhancer Activates Master Transcription Factor NR3C1 Expression and Promotes 5‐FU Resistance in Gastric Cancer,” Advanced Science 12, no. 7 (2024): 2409050. 39731339 10.1002/advs.202409050 PMC11831572

[52] Q. Jia , Y. Zhu , H. Yao , et al., “Oncogenic GALNT5 Confers FOLFIRINOX Resistance via Activating the MYH9/NOTCH/ DDR Axis in Pancreatic Ductal Adenocarcinoma,” Cell Death & Disease 15, no. 10 (2024): 1–14. 39433745 10.1038/s41419-024-07110-w PMC11493973

[53] K. M. Hanssen , M. Haber , and J. I. Fletcher , “Targeting Multidrug Resistance‐Associated Protein 1 (MRP1)‐Expressing Cancers: Beyond Pharmacological Inhibition,” Drug Resistance Updates 59 (2021): 100795. 34983733 10.1016/j.drup.2021.100795

[54] K. L. Aung , S. E. Fischer , R. E. Denroche , et al., “Genomics‐Driven Precision Medicine for Advanced Pancreatic Cancer: Early Results From the COMPASS Trial,” Clinical Cancer Research 24, no. 6 (2018): 1344–1354. 29288237 10.1158/1078-0432.CCR-17-2994 PMC5968824

[55] Z. Ni , P. Zhu , L. Liu , et al., “Single‐Cell Transcriptomic Analysis Reveals Epithelial‐Mesenchymal Transition and Key Gene AGRN as a Universal Programme in Gastrointestinal Tumours by an Artificial Intelligence‐Derived Prognostic Index,” Med Research (2025).

[56] Y. Zhao , Y. Zheng , Y. Zhu , et al., “M1 Macrophage‐Derived Exosomes Loaded With Gemcitabine and Deferasirox Against Chemoresistant Pancreatic Cancer,” Pharmaceutics 13, no. 9 (2021): 1493. 34575569 10.3390/pharmaceutics13091493 PMC8472397

[57] A. Vivancos , E. Aranda , M. Benavides , et al., “Comparison of the Clinical Sensitivity of the Idylla Platform and the OncoBEAM RAS CRC Assay for KRAS Mutation Detection in Liquid Biopsy Samples,” Scientific Reports 9, no. 1 (2019): 8976. 31222012 10.1038/s41598-019-45616-y PMC6586620

[58] K. Kataoka , T. Yamada , M. Shiozawa , et al., “Monitoring ctDNA RAS Mutational Status in Metastatic Colorectal Cancer: A Trial Protocol of RAS‐Trace and RAS‐Trace‐2 Studies,” Journal of the Anus, Rectum and Colon 8, no. 2 (2024): 132. 38689780 10.23922/jarc.2023-051 PMC11056539

[59] T. J. Morris , L. M. Butcher , A. Feber , et al., “ChAMP: 450k Chip Analysis Methylation Pipeline,” Bioinformatics 30, no. 3 (2014): 428–430. 24336642 10.1093/bioinformatics/btt684 PMC3904520

[60] A. E. Teschendorff , F. Marabita , M. Lechner , et al., “A Beta‐mixture Quantile Normalization Method for Correcting Probe Design Bias in Illumina Infinium 450 k DNA Methylation Data,” Bioinformatics 29, no. 2 (2013): 189–196. 23175756 10.1093/bioinformatics/bts680 PMC3546795

[61] Y. Li , D. Ge , and C. Lu , “The SMART App: An Interactive Web Application for Comprehensive DNA Methylation Analysis and Visualization,” Epigenetics & Chromatin 12, no. 1 (2019): 71. 31805986 10.1186/s13072-019-0316-3 PMC6894252

