# Supplementary material for: NR3C1, LAX1, and RCAN3 as Circulating Epigenetic Biomarkers for Prognosis and Chemotherapy Response Prediction in Metastatic Pancreatic Cancer
Source: MedComm (2020). 2026 Mar 22;7(4):e70682. doi: 10.1002/mco2.70682 (PMC13042955; doi:10.1002/mco2.70682)
Supplement: Supplementary file 1 — Figure S1: Comparative analysis of CpG methylation levels of KIAA1949 gene in plasma samples from healthy individuals and mPDAC patients. Figure S2: Residuals versus fitted values plots from multiple linear regression analyses evaluating the potential confounding effect of age on methylation levels for LAX1, RCAN3, and NR3C1 genes (model: methylation = group + age). Residuals were randomly distributed around zero, indicating no systematic bias and supporting that age does not significantly influence the observed group differences. Fitted values (X‐axis): predicted methylation levels estimated by the linear model (adjusted for group and age). Residuals (Y‐axis): the difference between the observed and predicted methylation values. p‐values correspond to the regression coefficient for age, indicating the lack of a significant association between age and methylation levels (p > 0.05). Figure S3: Correlation between basal circulating LAX1, RCAN3, and NR3C1 methylation levels with other circulating tumor biomarkers in mPDAC. LAX1, RCAN3, and NR3C1 methylation levels in plasma at diagnosis according to CA19‐9 levels, cfDNA concentration, cfDNA fragmentation and RAS MAF. Pearson's correlation p‐values and Benjamini–Hochberg false discovery rate (FDR)‐adjusted q‐values are shown. Figure S4: (A) cfDNA concentration and (B) cfDNA fragmentation in healthy individuals, compared with metastatic (stage IV) pancreatic cancer patients. Figure S5: Kaplan–Meier survival analyses of mPDAC patients stratified according to ROC curve‐derived cut‐off values for LAX1, RCAN3, and NR3C1 methylation markers. Overall survival (OS) and progression‐free survival (PFS) were compared between high‐ and low‐methylation groups using the log‐rank test. Results are consistent with those obtained using R2‐defined cut‐off values. Figure S6: Correlation between early changes of circulating biomarkers and progression‐free survival. Graphs show the slope from diagnosis to first follow‐up after treatment ini [file MCO2-7-e70682-s001.docx]

**SUPPLEMENTARY INFORMATION**

***NR3C1*, *LAX1* and *RCAN3* as circulating epigenetic biomarkers for prognosis and chemotherapy response prediction in metastatic pancreatic cancer.**

Cano-Ramírez P. et al.


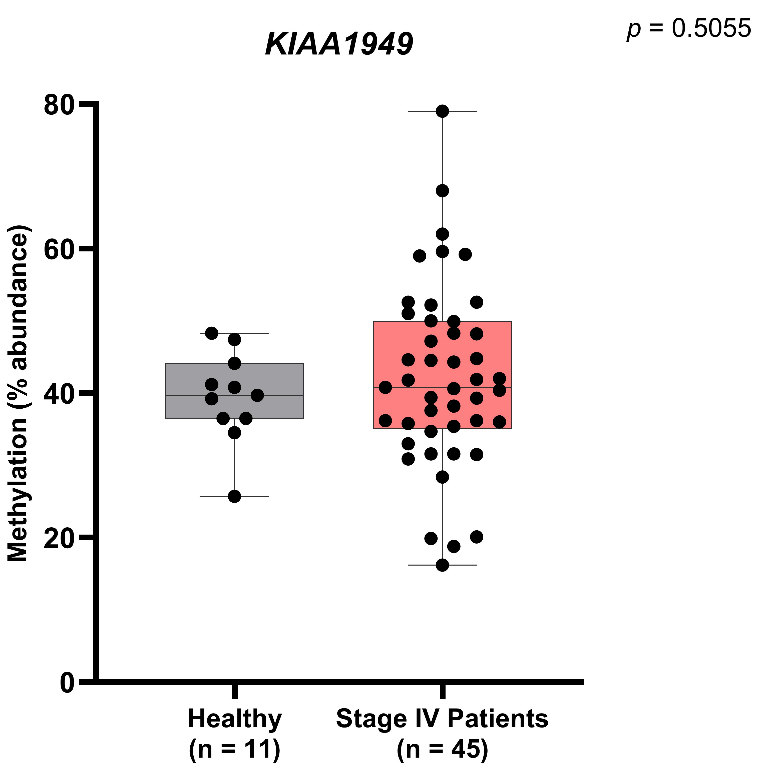


**Figure S1. Comparative analysis of CpG methylation levels of *KIAA1949* gene in plasma samples from healthy individuals and mPDAC patients.**

**Figure S2. Residuals *vs*. fitted values plots from multiple linear regression analyses evaluating the potential confounding effect of age on methylation levels for *LAX1*, *RCAN3*, and *NR3C1* genes** (model: methylation = group + age). Residuals were randomly distributed around zero, indicating no systematic bias and supporting that age does not significantly influence the observed group differences. Fitted values (X-axis): predicted methylation levels estimated by the linear model (adjusted for group and age). Residuals (Y-axis): the difference between the observed and predicted methylation values. *p*-values correspond to the regression coefficient for age, indicating the lack of a significant association between age and methylation levels (*p* > 0.05).

**Figure S3.** **Correlation between basal circulating *LAX1*, *RCAN3* and *NR3C1* methylation levels with other circulating tumor biomarkers in mPDAC.** *LAX1, RCAN3* and *NR3C1* methylation levels in plasma at diagnosis according to CA19-9 levels, cfDNA concentration, cfDNA fragmentation and *RAS* MAF. Pearson’s correlation *p*-values and Benjamini–Hochberg False Discovery Rate (FDR)-adjusted *q*-values are shown.


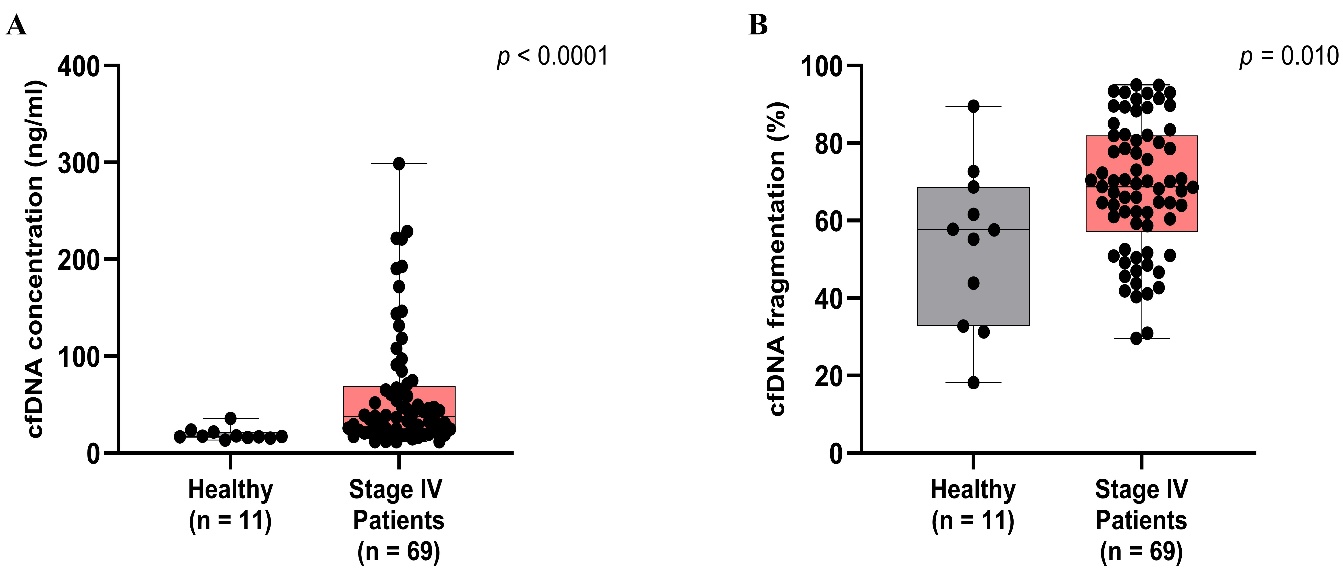


**A**

**B**

**Figure S4.** (**A**) **cfDNA concentration and (B) cfDNA fragmentation in healthy individuals compared with metastatic (stage IV) pancreatic cancer patients.**

**Figure S5. Kaplan–Meier survival analyses of mPDAC patients stratified according to ROC curve–derived cut-off values for *LAX1*, *RCAN3* and *NR3C1* methylation markers.** Overall survival (OS) and progression-free survival (PFS) were compared between high- and low-methylation groups using the log-rank test. Results are consistent with those obtained using R2-defined cut-off values.

**Figure S6. Correlation between early changes of circulating biomarkers and progression-free survival**. Graphs show the slope from diagnosis to first follow-up after treatment initiation of *LAX1* methylation, *RCAN3* methylation, *NR3C1* methylation, CA19-9 levels, *RAS* mutant allele fraction, cfDNA concentration and cfDNA fragmentation.

**
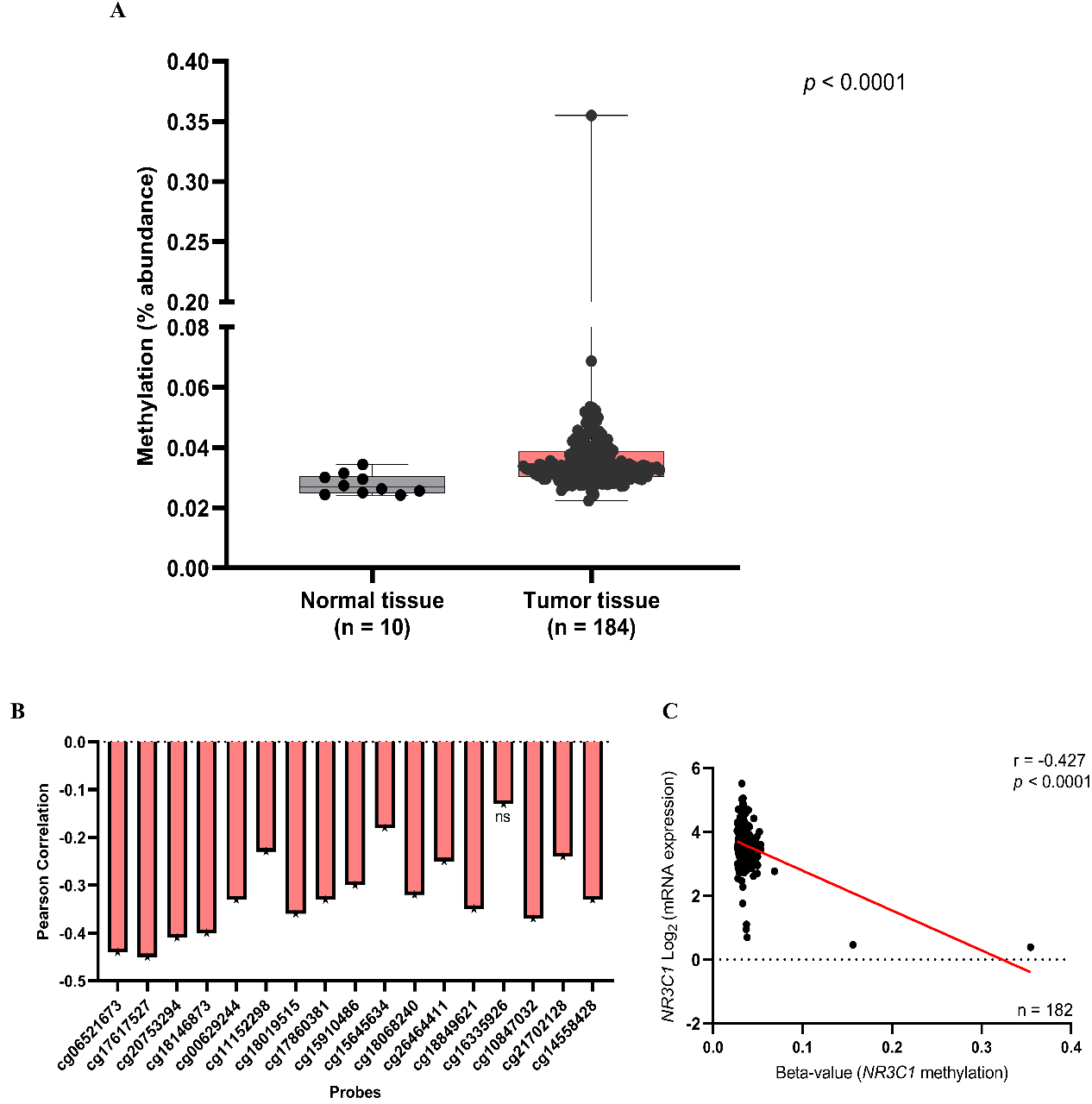
**

**A**

**
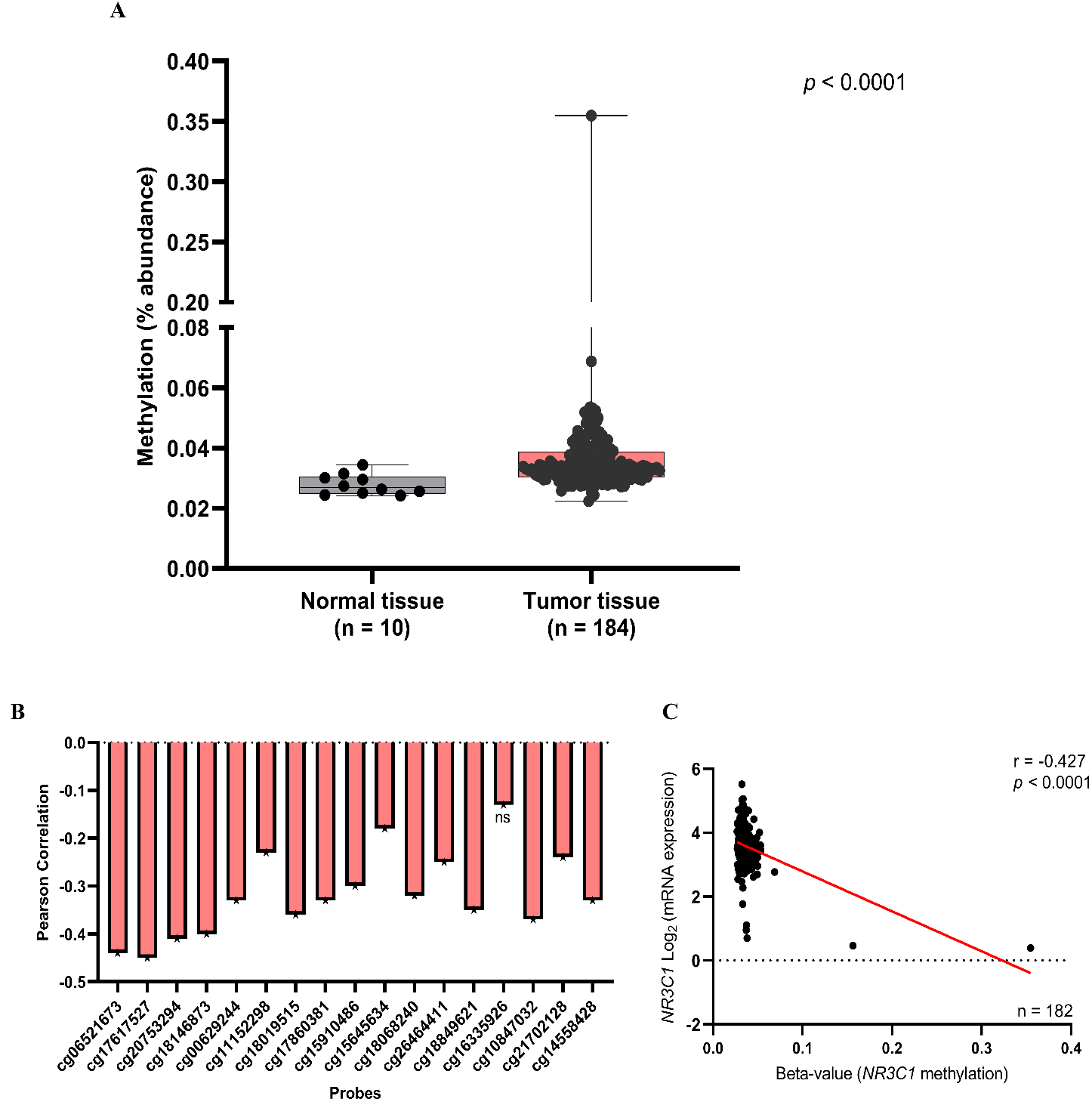
**

**C**

**B**

**Figure S7. Aberrant methylation of the *NR3C1* promoter contributes to its transcriptional downregulation in PDAC.** (**A**) Average methylation of 17 CpGs sites within the proximal promoter CpG island in normal tissue and PDAC tumor tissue samples. (**B**) Pearson correlation coefficient for each CpG position. (**C**) Correlation between global proximal promoter CpG island methylation and *NR3C1* mRNA levels. All data were from The Cancer Genome Atlas (TCGA) database.

**Table S1.** Methylation levels of 9 CpG positions (adjusted *p*-value ≤ 0.05; Δβ ≥ 0.20) with negative and positive controls of methylation. ± SD indicate Poisson’s error (95% CI).

| **GENE** | **Negative control** | | **Positive control** | |
| --- | --- | --- | --- | --- |
|  | Droplets | Methylation  (% abundance)  ± Poisson Error | Droplets | Methylation  (% abundance)  ± Poisson Error |
| *THOP1* | 17025 | 29.1 ± 4.2 | 880 | __ |
| *SPPL2B* | 17698 | 34.1 ± 5.1 | 12602 | 100 ± 0.5 |
| *TCF7* | 19098 | 3.96 ± 1.8 | 13350 | 93.2 ± 2.9 |
| *MGAT4A* | 18194 | 29.5 ± 2.9 | 14465 | 98.4 ± 0.8 |
| ***NR3C1*** | 17248 | **4.8 ± 1.5** | 14255 | **96.9 ± 0.9** |
| ***KIAA1949*** | 16909 | **3.14 ± 1.34** | 14140 | **98.1 ± 1** |
| ***LAX1*** | 17535 | **0.654 ± 0.8** | 14357 | **97.2 ± 1.7** |
| ***RCAN3*** | 17906 | **4.53 ± 1.9** | 12487 | **98.7 ± 0.7** |
| *STIM2* | 18048 | 6.12 ± 2.77 | 12265 | 100 ± 0.9 |

**Table S2.** Multivariable Cox regression analysis for overall survival and progression free survival according to basal circulating biomarkers in metastatic PDAC patients.

| **Variable** | **OS** | | **PFS** | |
| --- | --- | --- | --- | --- |
|  | **HR (95% CI)** | ***p*** | **HR (95% CI)** | ***p*** |
| **CA19-9** |  |  |  |  |
| > 4723 (U/mL)^R^ | 2.150 | 0.012 | 2.932 | 0.001 |
| ≤ 4723 (U/mL) | (1.180 - 3.906) |  | (1.574 – 5.464) |  |
| ***RAS* status in plasma** |  |  |  |  |
| *RAS* mutated^R^ | - | *ns* | 4.347 | 0.001 |
| *RAS* wild-type |  |  | (1.792 – 10.526) |  |
| **cfDNA fragmentation** |  |  |  |  |
| > 75.82 %ᴿ | - | *ns* | 2.531 | 0.006 |
| ≤ 75.82 % |  |  | (1.303 – 4.901) |  |
| ***LAX1* methylation** |  |  |  |  |
| > 92.20 %ᴿ | - | *ns* | 1.934 | 0.044 |
| ≤ 92.20 % |  |  | (1.019 - 3.676) |  |
| ***NR3C1* methylation** |  |  |  |  |
| > 90.40 %ᴿ | 0.546 | 0.05 | - | *ns* |
| ≤ 90.40 % | (0.302 - 1.006) |  |  |  |
| **ECOG** |  |  |  |  |
| 0ᴿ |  |  |  |  |
| 1 | - | *ns* | 3.745 | 0.003 |
|  |  |  | (1.557 – 9.009) |  |
| 2 or 3 | - | *ns* | 3.278 | 0.013 |
|  |  |  | (1.283 – 8.403) |  |
| **First-line treatment** |  |  |  |  |
| FOLFIRINOX-basedᴿ | 0.347 | 0.001 | - | *ns* |
| Gemcitabine-based | (0.185 – 0.649) |  |  |  |

^R^ Reference category for analysis.

**Table S3.** Primers and probes for methylation analysis of different genes in cfDNA.

| **Target** | **Name** | | **Sequence 5'-3'** |
| --- | --- | --- | --- |
| *NR3C1* | | Forward | GTGTTTTTATGTTGTTGTTTAAAAGATGGG |
|  |  | Reverse | CATAAACCAAAATCACACCACTACAC |
|  |  | Probe M | /56-FAM/TGAGACGAG/ZEN/TTTTATTTTGTTATTTAGGT/3IABkFQ/ |
|  |  | Probe U | /5SUN/TGAGATGAG/ZEN/TTTTATTTTGTTATTTAGGT/3IABkFQ/ |
| *RCAN3* | | Forward | GTTGTGAGGTTTTTGGTTGTGG |
|  |  | Reverse-R | ACAAAATCTCACCTATACTATTC**R**CAAC |
|  |  | Probe M | /56-FAM/ATGGCGTTT/ZEN/TGTGTTTGCGGTCGATGC/3IABkFQ/ |
|  |  | Probe U | /5SUN/ATGGTGTTT/ZEN/TGTGTTTGTGGTTGATGTGT/3IABkFQ/ |
| *LAX1* | | Forward | GTGAAGGTAGAGGTTATAGATTTTTT |
|  |  | Reverse | CCTAAACGTAACTAATACCCACTA |
|  |  | Probe M^(AP)^ | /56-FAM/CG+GATGAG+A+T+C+GTAT+TT/3IABkFQ/ |
|  |  | Probe U^(AP)^ | /5SUN/TGGA+T+GAGA+T+T+GT+AT/3IABkFQ/ |
| *KIAA1949* | | Forward | GTAGGAGTGGAGGTAGTAGAG |
|  |  | Reverse | TCCATTTCCACCCTTCTATTAACT |
|  |  | Probe M^(AP)^ | /56-FAM/AAG+ATG+G+C+GAG+AG/3IABkFQ/ |
|  |  | Probe U^(AP)^ | /5SUN/AG+AT+G+G+T+GA+GA/3IABkFQ/ |

^(AP)^Affinity Plus™
